# Safety and Differential Antibody and T-Cell Responses to the *Plasmodium falciparum* Sporozoite Malaria Vaccine, PfSPZ Vaccine, by Age in Tanzanian Adults, Adolescents, Children, and Infants

**DOI:** 10.4269/ajtmh.18-0835

**Published:** 2019-04-15

**Authors:** Said A. Jongo, L. W. Preston Church, Ali T. Mtoro, Sumana Chakravarty, Adam J. Ruben, Phillip A. Swanson, Kamaka R. Kassim, Maximillian Mpina, Anneth-Mwasi Tumbo, Florence A. Milando, Munira Qassim, Omar A. Juma, Bakari M. Bakari, Beatus Simon, Eric R. James, Yonas Abebe, Natasha KC, Elizabeth Saverino, Linda Gondwe, Fabian Studer, Martina Fink, Glenda Cosi, Jill El-Khorazaty, David Styers, Robert A. Seder, Tobias Schindler, Peter F. Billingsley, Claudia Daubenberger, B. Kim Lee Sim, Marcel Tanner, Thomas L. Richie, Salim Abdulla, Stephen L. Hoffman

**Affiliations:** 1 Ifakara Health Institute, Bagamoyo Research and Training Centre, Bagamoyo, Tanzania;; 2 Sanaria, Inc., Rockville, Maryland;; 3 Vaccine Research Center (VRC), National Institute of Allergy and Infectious Diseases, National Institutes of Health, Bethesda, Maryland;; 4 Swiss Tropical and Public Health Institute, Basel, Switzerland;; 5 University of Basel, Basel, Switzerland;; 6 The Emmes Corporation, Rockville, Maryland;; 7 Protein Potential LLC, Rockville, Maryland

## Abstract

In 2016, there were more cases and deaths caused by malaria globally than in 2015. An effective vaccine would be an ideal additional tool for reducing malaria’s impact. Sanaria^®^ PfSPZ Vaccine, composed of radiation-attenuated, aseptic, purified, cryopreserved *Plasmodium falciparum* (Pf) sporozoites (SPZ) has been well tolerated and safe in malaria-naïve and experienced adults in the United States and Mali and protective against controlled human malaria infection with Pf in the United States and field transmission of Pf in Mali, but had not been assessed in younger age groups. We, therefore, evaluated PfSPZ Vaccine in 93 Tanzanians aged 45 years to 6 months in a randomized, double-blind, normal saline placebo-controlled trial. There were no significant differences in adverse events between vaccinees and controls or between dosage regimens. Because all age groups received three doses of 9.0 × 10^5^ PfSPZ of PfSPZ Vaccine, immune responses were compared at this dosage. Median antibody responses against Pf circumsporozoite protein and PfSPZ were highest in infants and lowest in adults. T-cell responses were highest in 6–10-year olds after one dose and 1–5-year olds after three doses; infants had no significant positive T-cell responses. The safety data were used to support initiation of trials in > 300 infants in Kenya and Equatorial Guinea. Because PfSPZ Vaccine–induced protection is thought to be mediated by T cells, the T-cell data suggest PfSPZ Vaccine may be more protective in children than in adults, whereas infants may not be immunologically mature enough to respond to the PfSPZ Vaccine immunization regimen assessed.

## INTRODUCTION

Despite an annual investment of more than $2.7 billion in insecticide-treated bed nets, indoor residual spraying, diagnosis, and treatment, in 2015, 2016, and 2017 there were an estimated 429,000–730,500 deaths each year caused by malaria^1–3^; 90% of the mortality was in children under the age of 5 years. *Plasmodium falciparum* (Pf) was the cause of more than 98% of deaths from malaria and more than 80% of cases of malaria in sub-Saharan Africa. Our goal is to field a vaccine that will prevent infection with Pf and thereby prevent all clinical and pathological manifestations of malaria and halt parasite transmission from humans to mosquitoes.^4^

A number of malaria vaccines are under development, but none have received marketing authorization (licensing) by a regulatory authority. RTS,S/AS01 has completed Phase 3 clinical trials,^5^ received a positive opinion (Article 58) from the European Medicines Agency^6^ and in 2019, large-scale pilot implementation trials will be initiated in Kenya, Malawi, and Ghana to confirm the level of protective efficacy, demonstrate that the entire immunization regimen can be successfully administered, and assess several safety signals seen in the Phase 3 trial (increased meningitis, febrile seizures, and female mortality in vaccinees as compared with controls).^7,8^ A second pre-erthrocytic stage vaccine ChAd63 and MVA ME-TRAP has also been studied in African infants to adults.^9–11^

Sanaria^®^ PfSPZ Vaccine is composed of radiation-attenuated, aseptic, purified, and cryopreserved *Plasmodium falciparum* (Pf) sporozoites (SPZ).^12^ The vaccine has been extremely well tolerated and safe in multiple clinical trials.^13–18^ In Mali, Equatorial Guinea, and Tanzania, there was no difference in adverse events (AEs) between the PfSPZ Vaccine and normal saline (NS) control in double-blind, placebo-controlled trials.^18–20^

PfSPZ Vaccine has been reported in malaria-naïve adults to have a vaccine efficacy (VE) of > 90% against controlled human malaria infection (CHMI) with homologous Pf parasites (same Pf strain in vaccine and CHMI),^14,16^ 80% against CHMI with heterologous Pf parasites (different Pf strain in vaccine and CHMI) 3 weeks after the last vaccine dose,^14,16^ 65% and 55% against homologous CHMI 24,^16^ and 59^15^ weeks and 54% against heterologous CHMI 33 weeks after the last vaccine dose.^17^ In Malian adults, VE against Pf infection during the 24 weeks after last vaccine dose was 52% by time to infection analysis and 29% by proportional analysis.^18^ Protection by immunization with sporozoites is dependent on T cells in mice and nonhuman primates^13,21–24^ and thought to be T cell–dependent in humans.^13^ The durable protection demonstrated in the Mali trial was associated with elevated gamma delta T-cell frequencies, providing support for this hypothesis.^25^

However, in Tanzanian adults, five doses of 2.7 × 10^6^ PfSPZ had a VE against 3- and 24-week homologous CHMI of 20%.^20^ This was the same immunization regimen used in the Mali trial that gave 52% VE and in a trial in the United States that gave 92% and 65% VE against 3- and 24-week homologous CHMI.^16^ In Tanzania, the antibody and T-cell responses to PfSPZ in adults were significantly lower than in adults in the United States^20^; antibody responses in Mali were even lower.^18^ We hypothesized that the lower immune responses in malaria-exposed African subjects as compared with malaria-naïve U.S. subjects were due to immune dysregulation caused by long-term exposure to malaria parasites^18,20^ and that naturally acquired immunity may have reduced the effective PfSPZ inoculum. We, therefore, proposed that injecting larger doses of PfSPZ might partially overcome these effects. This is in part because when humans are immunized with radiation-attenuated PfSPZ administered by mosquito bite,^26^ PfSPZ Vaccine^14,15,17,27^ and PfSPZ-CVac^27^ protection is dose dependent.^26^ Thus, increasing immune responses by increasing the dose should increase VE. Thus, in this study, we increased the dose of PfSPZ Vaccine from 2.7 × 10^5^ PfSPZ to 9.0 × 10^5^ PfSPZ and 1.8 × 10^6^ PfSPZ.

All previous studies of PfSPZ Vaccine have been conducted in adults. However, the major burden of malaria is in older infants and children. The present study was the first to assess the tolerability, safety, and immunogenicity of PfSPZ Vaccine in adolescents, children, and infants aged 6 months and older and the first to compare these results with those of adults. Furthermore, we hypothesized that infants and young children with little previous exposure to Pf parasites would have more robust immune responses to the vaccine than adults, recognizing that the infants’ immunological systems might not be fully mature, particularly for T-cell responses.^28^

## MATERIAL AND METHODS

### Study design and population.

This single-center, age de-escalation, double-blind, randomized, placebo-controlled trial (ClinicalTrials registration no. NCT02613520) was conducted in Bagamoyo, Tanzania, between December 2015 and March 2017. It had two major components, an age de-escalation, dose escalation component to assess safety, tolerability, and immunogenicity of PfSPZ Vaccine (part A), and a CHMI component to assess VE (part B). Herein, we report the results of part A.

One hundred seventy-three healthy male and female volunteers aged 6 months to 45 years were recruited from the Bagamoyo region through locally presented sensitization meetings. After an initial screening, prospective volunteers were invited to the Bagamoyo Clinical Trial Unit (BCTU) of the Ifakara Health Institute (IHI) to complete the informed consent process and further screening.

Informed consent was obtained from all volunteers or the parents/legal guardians after the nature and risks of the study were explained. Following this, the adult volunteers or the parent/legal guardian of child volunteers were required to complete a 10-question assessment with a 100% correct response rate on the first or second attempt to demonstrate understanding of the study procedures (Supplemental Table 1) to be eligible for enrollment. In addition, all children and adolescents aged 9–18 years provided written assent and children aged 6–8 years provided oral assent. Volunteers were screened using predetermined inclusion and exclusion criteria based on clinical examinations and laboratory tests (Supplemental Tables 2 and 3). Medical history was analyzed to exclude any past or present medical problem in conjunction with a detailed clinical examination. Laboratory testing included hematology, biochemistry, urinalysis, and parasitology testing to include malaria thick blood smear (TBS), stool for intestinal helminth infections, and urine for *Schistosoma haematobium*. Tests for HIV and hepatitis B and C were performed only after pretest counseling was carried out; volunteers were excluded if positive and referred for further evaluation and management. Volunteers were excluded if they had significant abnormalities on electrocardiograms. The complete eligibility criteria are published at https://clinicaltrials.gov/show/NCT02613520.

The trial was performed in accordance with good clinical practices. The protocol was approved by institutional review boards (IRBs) of the IHI (Ref. No. IHI/IRB/ No: 32-2015), the National Institute for Medical Research Tanzania (NIMR/HQ/R.8a/Vol.IX/2049), and the Ethikkommission Nordwest- und Zentralschweiz, Basel, Switzerland (reference number 15/104). The protocol was also approved by the Tanzania Food and Drug Authority (Auth. No. TZ15CT013), registered at ClinicalTrials.gov (NCT02613520) and conducted under a U.S. Food and Drug Administration Investigational New Drug application (FDA IND) application.

### Intervention and randomization.

Volunteers spanning five age groups were sequentially allocated to 11 different dose groups and randomly assigned to receive PfSPZ Vaccine or NS in a 2:1 ratio. Twelve additional, age-matched, adult volunteers were enrolled as nonimmunized infectivity controls for CHMI studies.

The details of each of five main age groups (Groups 1–5) are outlined in Table 1. Immunization began with the adults (Group 1a, 9.0 × 10^5^ PfSPZ and Group 1b, 1.8 × 10^6^ PfSPZ) and continued progressively to teenagers (11–17 years; Group 2a, 9.0 × 10^5^ PfSPZ and Group 2b, 1.8 × 10^6^ PfSPZ), older children (6–10 years; Group 3a, 9.0 × 10^5^ PfSPZ and Group 3b, 1.8 × 10^6^ PfSPZ), younger children (1–5 years; Group 4a, 4.5 × 10^5^ PfSPZ and Group 4b, 9.0 × 10^5^ PfSPZ), and infants (6–11 months; Group 5a, 2.7 × 10^5^ PfSPZ, Group 5b, 4.5 × 10^5^ PfSPZ, and Group 5c, 9.0 × 10^5^ PfSPZ). Only after the safety of a given PfSPZ dose had been demonstrated in an older age group was the same or a lower dose tested next in a younger age group (age de-escalation). Likewise, within each age group, safety was demonstrated with a lower PfSPZ dose before immunizations began with a higher PfSPZ (dose escalation). At three time points during age de-escalation and dose escalation, a three-member external Safety Monitoring Committee reviewed safety reports and provided a recommendation to proceed to the next study group.

**Table 1:** Vaccine and control groups by age, vaccine dose, dosing schedule, number of doses, and total number of *Plasmodium falciparum* sporozoites (PfSPZ)

Groups	Age (years)	Subgroups details		*N*	PfSPZ/dose	Dosing schedule (weeks)	No. doses	Total PfSPZ
Group 1	18–45	1a	Vaccinees	6	9 × 10^5^	0, 8, 16	3	2.7 × 10^6^
			NS controls	3	NS	0, 8, 16	3	0
		1b	Vaccinees	6	1.8 × 10^6^	0, 8, 16	3	5.4 × 10^6^
			NS controls	3	NS	0, 8, 16	3	0
Group 2	11–17	2a	Vaccinees	6	9 × 10^5^	0, 8, 16	3	2.7 × 10^6^
			NS controls	3	NS	0, 8, 16	3	0
		2b	Vaccinees	6	1.8 × 10^6^	0, 8, 16	3	5.4 × 10^6^
			NS controls	3	NS	0, 8, 16	3	0
Group 3	6–10	3a	Vaccinees	6	9 × 10^5^	0, 8, 16	3	2.7 × 10^6^
			NS controls	3	NS	0, 8, 16	3	0
		3b	Vaccinees	6	1.8 × 10^6^	0, 8, 16	3	5.4 × 10^6^
			NS controls	3	NS	0, 8, 16	3	0
Group 4	1–5	4a	Vaccinees	6	4.5 × 10^5^	0, 8, 16	3	1.35 × 10^6^
			NS controls	3	NS	0, 8, 16	3	0
		4b	Vaccinees	6	9.0 × 10^5^	0, 8, 16	3	2.7 × 10^6^
			NS controls	3	NS	0, 8, 16	3	0
Group 5	6–11 months	5a	Vaccinees	3	2.7 × 10^5^	0	1	2.7 × 10^5^
		5b	Vaccinees	6	4.5 × 10^5^	0, 8, 16	3	1.35 × 10^6^
			NS controls	3	NS	0, 8, 16	3	0
		5c	Vaccinees	6	9 × 10^5^	0, 8, 16	3	2.7 × 10^6^
			NS controls	3	NS	0, 8, 16	3	0
Total				93				

NS = normal saline.

Using this staggered approach, 10 subgroups of nine individuals received three doses of PfSPZ Vaccine at 56-day intervals (days 1, 57, and 113), each comprising six vaccine and three placebo volunteers randomized in a 2:1 ratio to PfSPZ Vaccine or NS control. An exception was Group 5a (Table 1), an 11th subgroup comprising three infants constituting the first ever recipients less than 1 year of age to receive injections with PfSPZ Vaccine. This safety-only pilot group was administered a single reduced dose of PfSPZ Vaccine (2.7 × 10^5^ PfSPZ); there was no randomization and no placebo control. All of the other 90 volunteers and the entire clinical team excluding the pharmacy staff were blinded to treatment assignment, with blinded status maintained throughout the study period.

### Investigational product (IP).

The IP used in this trial, Sanaria PfSPZ Vaccine,^12–19^ consists of aseptic, purified, vialed, metabolically active, nonreplicating (live, radiation attenuated) cryopreserved PfSPZ stored in liquid nitrogen vapor phase at −150 to −196°C. Preparation of IP was done under the supervision of the study pharmacist, who was not blinded to the study treatment for each volunteer. Vials of PfSPZ Vaccine were thawed and diluted with phosphate-buffered saline containing human serum albumin and the appropriate numbers of PfSPZ in a final volume of 0.5 mL and drawn into a 1-mL syringe. A volume of 0.5 mL of NS was similarly drawn into a 1-mL syringe for placebo recipients. Dilution and syringe preparation were performed under aseptic conditions in a biological safety cabinet. The pharmacist then handed the appropriate syringe for the specific study subject to the blinded nurse through a window. Reconstituted PfSPZ is a clear, odorless, nonviscous liquid indistinguishable from NS. PfSPZ Vaccine or NS was administered by direct venous inoculation (DVI) through a 25 G × 16-mm needle. In infants and young children, there was an option to administer the vaccine through a 24-gauge peripheral intravenous catheter.

### Assessment of vaccine safety, tolerability of DVI, and AEs.

After vaccination, volunteers under the age of 18 years were observed at the BCTU for approximately 24 hours during which enquiry of AEs and focused physical examinations were performed at 1, 6, and 12 hours after vaccination and at the time of discharge. Adult volunteers were observed at the BCTU for approximately 2 hours during which enquiry of AEs and focused physical examinations were carried out at 1 hour after vaccination and at the time of discharge. Volunteers were given diaries and thermometers for recording of AEs and temperatures thereafter. Volunteers were seen for safety follow-up visits 2, 7, and 14 days after vaccination, with additional follow-up visits by telephone on days 3–6 (and Day 1 for adults). Children and infants were also evaluated again 28 and 56 days after the final immunization.

Local (site of injection) signs and symptoms were solicited in the 2 days following vaccination for adults, teenagers, and older children (groups 1–3) or 7 days following vaccination for younger children and infants (groups 4 and 5). Systemic signs and symptoms were assessed for 7 days for all groups (Supplemental Table 4). In addition, open-ended questioning was used to identify unsolicited AEs through day 28 postimmunization.

During the period of follow-up, all solicited and unsolicited events were recorded and graded by the attending physician as follows: mild (no effect on activities), moderate (some interference with normal activity), severe (prevented normal activity and required medical intervention), life-threatening (hospitalization, immediate medical intervention, or therapy required to prevent death), or death. Axillary temperature was recorded as Grade 1 (> 37.9–38.4°C), Grade 2 (> 38.4–38.9°C), Grade 3 (> 38.9–40.0°C), or Grade 4 (> 40.0°C). Hematological and biochemical abnormalities were also assessed at prespecified intervals as defined in the toxicity table of the study protocol, including prevaccination and 7 days postvaccination.

### Malaria parasite diagnosis.

All participants were screened for malaria parasites at baseline and before vaccination by TBS microscopy and retrospectively by quantitative polymerase chain reaction (qPCR). Slide preparation and reading for TBS’s were performed according to standard procedures.^29^ The theoretical limit of detection of TBS was 2 parasites/µL (0.5 µL blood examined) for standard reads and 0.5 parasites/µL (2.0 µL blood examined) for expanded reads done when a volunteer was symptomatic. Quantitative polymerase chain reaction analyses were based on DNA extracted from 180 µL whole blood and amplification of the *pan-Plasmodium* 18S gene^30^ and the Pf-specific telomere-associated repetitive element 2^31^ following essentially the published procedures. The 18S gene DNA qPCR had a sensitivity of 50 parasites/mL. *Plasmodium malariae* (Pm) cases were identified by a qPCR species identification assay using Pm-specific amplification of plasmepsin 4 as described.^32^

#### Genotyping of parasites.

DNA from Pf-positive samples was used to genotype the parasites based on Pf Merozoite Surface Protein-1 (PfMSP-1) and PfMSP-2 gene polymorphisms^33^ as well as seven microsatellite markers.^34^ All Pf strains were compared with the Pf vaccine strain (NF54).

### Antibody assays.

Sera were assessed for antibodies by enzyme-linked immunosorbent assay (ELISA) to the major protein on the surface of sporozoites (Pf circumsporozoite protein [PfCSP]), immunofluorescence assay (aIFA) to air-dried PfSPZ, and inhibition of sporozoite (PfSPZ) invasion (aISI) of HC-04 cells (hepatocytes) as described.^27^

### T-cell assays.

T-cell responses in cryopreserved peripheral blood mononuclear cells (PBMCs) were assessed by flow cytometry as described.^15^

### Statistical analysis.

The sample sizes of three to six vaccinees in each age group dosage group, 12–15 vaccinees in each age category, and six controls in each age category were selected to be appropriate for the initial assessment of safety, tolerance, and immunogenicity of an investigational vaccine. Categorical variables were summarized using absolute (*n*) and relative (%) frequencies. Continuous variables were summarized using mean and SD, median, and range. Comparisons of categorical variables between groups were analyzed using Barnard’s two-sided exact unconditional test, or a two-sided Mantel–Haenszel test stratifying by age group. No corrections were made for multiple comparisons because of the early phase nature of this trial. Analyses of immunological data are described with the data. A *P* value < 0.05 was considered significant. All data analyses and statistical computations were conducted with SAS software, version 9.3 or higher (SAS Institute, Inc., Cary, NC) or GraphPad Prism, version 7.02 (GraphPad Software, LaJolla, CA).

## RESULTS

### Study population, experience with DVI, and tolerability.

**Figure 1.**
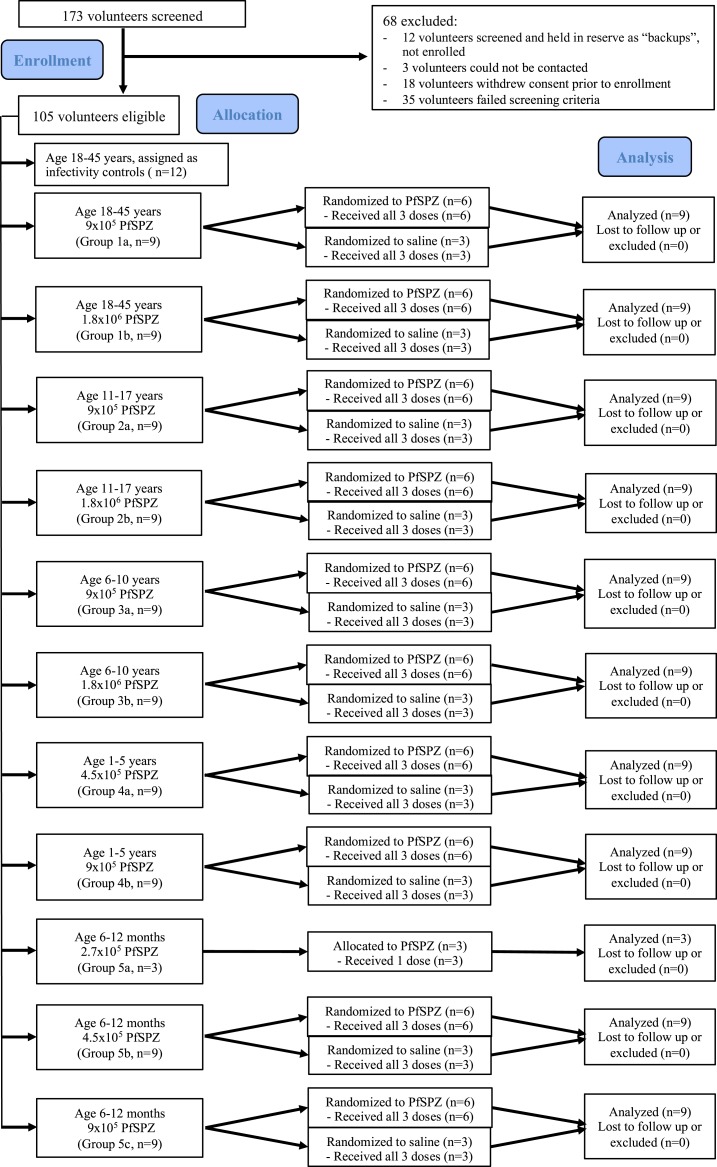
Volunteer participation (CONSORT 2010 Diagram). Once allocated, no volunteers were removed, lost to follow-up, or excluded from analysis. This figure appears in color at www.ajtmh.org.

A total of 105 Tanzanian volunteers (Figure 1) met the criteria (Supplemental Tables 2 and 3) and were enrolled. Ninety-three received either PfSPZ Vaccine (*n* = 63) or NS (*n* = 30) (Table 1). The remaining 12 volunteers participated as infectivity controls in the subsequent challenge portion (CHMI) of the protocol and are not part of the analyses in this article. There were no significant differences between participants in any age group for age, height, weight, or body mass index (BMI) (*P* > 0.05 for all comparisons, one-sided analysis of variance (ANOVA) (Table 2). All 93 volunteers received all scheduled immunizations. One volunteer in group 5b received only a partial vaccine dose with the second immunization; all other volunteers received the complete 0.5-mL injection at all time points.

**Table 2:** Volunteer characteristics

		Group 1 (18–45 years)				Group 2 (11–17 years)			Group 3 (6–10 years)		
		9 × 10^5^ (*N* = 6)	1.8 × 10^6^ (*N* = 6)	Placebo (*N* = 6)	CHMI controls (*N* = 12)	9 × 10^5^ (*N* = 6)	1.8 × 10^6^ (*N* = 6)	Placebo (*N* = 6)	9 × 10^5^ (*N* = 6)	1.8 × 10^6^ (*N* = 6)	Placebo (*N* = 6)
Age	Units	Years	Years	Years	Years	Years	Years	Years	Years	Years	Years
	Mean (SD)	23.5 (5.7)	24.2 (5.3)	28.7 (7.9)	23.9 (4.8)	11.8 (1.0)	13.2 (1.3)	12.2 (1.3)	7.8 (1.5)	7.8 (1.5)	7.8 (1.5)
	Median	22	24	30	23	12	13	12	8	8	8
	(min, max)	(20, 35)	(18, 33)	(19, 38)	(18, 36)	(11, 13)	(11, 15)	(11, 14)	(6, 10)	(6, 10)	(6, 10)
Sex	Male	4 (66.7%)	4 (66.7%)	5 (83.3%)	7 (58.3%)	3 (50.0%)	2 (33.3%)	3 (50.0%)	3 (50.0%)	4 (66.7%)	3 (50.0%)
	Female	2 (33.3%)	2 (33.3%)	1 (16.7%)	5 (41.7%)	3 (50.0%)	4 (66.7%)	3 (50.0%)	3 (50.0%)	2 (33.3%)	3 (50.0%)
Race	African	6 (100%)	6 (100%)	6 (100%)	12 (100%)	6 (100%)	6 (100%)	6 (100%)	6 (100%)	6 (100%)	6 (100%)
Height (cm)	Mean (SD)	163.2 (5.0)	166.5 (10.4)	166.3 (8.0)	157.1 (9.0)	139.9 (15.3)	153.5 (9.8)	145.4 (9.2)	123.2 (9.2)	122.0 (7.8)	123.3 (7.1)
	Median	164.0	166.0	166.0	158.3	139.8	154.3	149.8	125.3	120.8	123.0
	(min, max)	(154, 168)	(149, 178)	(153, 175)	(136, 167)	(118, 165)	(138, 165)	(134, 154)	(112, 137)	(114, 135)	(114, 135)
Weight (kg)	Mean (SD)	62.3 (8.1)	65.8 (11.2)	64.3 (3.3)	58.0 (8.4)	33.2 (10.9)	40.6 (9.4)	37.8 (9.3)	23.3 (5.4)	21.2 (4.5)	22.9 (2.4)
	Median	62.5	67.0	65.0	57.5	31.5	41.0	39.3	24.0	20.5	22.5
	(min, max)	(53, 70)	(51, 80)	(60, 68)	(44, 75)	(21, 53)	(28, 55)	(25, 51)	(17, 29)	(16, 29)	(20, 27)
BMI	Mean (SD)	23.6 (4.1)	23.7 (2.9)	23.4 (2.6)	23.6 (3.8)	16.6 (2.6)	17 (2.4)	17.6 (2.7)	15.2 (1.9)	14.1 (1.2)	15.1 (.7)
	Median	23.5	23.6	23.1	23.4	16.1	16.5	17.3	15.1	14.1	15.2
	(min, max)	(19, 30)	(20, 28)	(20, 27)	(19, 31)	(14, 20)	(15, 20)	(14, 22)	(13, 18)	(12, 16)	(14, 16)

		Group 4 (1–5 years)			Group 5 (6–11 months)			
		4.5 × 10^5^ (*N* = 6)	9 × 10^5^ (*N* = 6)	Placebo (*N* = 6)	2.7 × 10^5^ (*N* = 3)	4.5 × 10^5^ (*N* = 6)	9 × 10^5^ (*N* = 6)	Placebo (*N* = 6)
Age	Units	Years	Years	Years	Months	Months	Months	Months
	Mean (SD)	3.3 (1.0)	2.7 (1.9)	3.0 (1.3)	7.3 (1.5)	7.3 (0.8)	8.7 (1.5)	9.3 (1.9)
	Median	3	3	3	7	8	8	10
	(min, max)	(2, 5)	(1, 5)	(2, 5)	(6, 9)	(6, 8)	(7, 11)	(6, 11)
Sex	Male	2 (33.3%)	3 (50.0%)	3 (50.0%)	1 (33.3%)	4 (66.7%)	2 (33.3%)	1 (16.7%)
	Female	4 (66.7%)	3 (50.0%)	3 (50.0%)	2 (66.7%)	2 (33.3%)	4 (66.7%)	5 (83.3%)
Race	African	6 (100%)	6 (100%)	6 (100%)	3 (100%)	6 (100%)	6 (100%)	6 (100%)
Height (cm)	Mean (SD)	99.7 (7.4)	91.6 (16.0)	96.1 (7.1)	66.0 (2.6)	67.7 (2.5)	69.9 (3.8)	66.0 (4.2)
	Median	99.3	92.0	93.8	65.0	68.3	71.8	66.5
	(min, max)	(92, 111)	(72, 113)	(89, 107)	(64, 69)	(63, 70)	(63, 73)	(59, 71)
Weight (kg)	Mean (SD)	14.3 (1.6)	12.3 (3.9)	13.3 (1.4)	7.0 (1.0)	8.0 (0.9)	8.4 (1.2)	7.8 (1.2)
	Median	14.5	11.3	13.0	7.0	8.0	8.8	7.5
	(min, max)	(12, 16)	(9, 18)	(12, 15)	(6, 8)	(7, 9)	(7, 10)	(7, 10)
BMI	Mean (SD)	14.5 (1.4)	14.5 (1.3)	14.5 (1.4)	16.0 (1.2)	17.5 (2.4)	17.2 (1.6)	18 (2.8)
	Median	14.6	14.4	14.5	16.6	17.9	17.2	16.9
	(min, max)	(13, 16)	(13, 17)	(13, 16)	(15, 17)	(14, 20)	(15, 20)	(16, 23)

CHMI = controlled human malaria infection.

Of 273 total injections, 234 were completed with a single injection (85.7%); 225 of these (96.2%) were assessed as simple to perform by the nurse performing the injection. In volunteers aged ≤ 5 years old, DVI was successful on the first attempt in 25 of 39 for first dose, 26 of 36 for second dose, and 31 of 36 for the third dose. The option to establish intravenous access with an intravascular catheter was used for 12 first injections in volunteers ≤ 5 years old, and one first injection in a 6–10-year old. An intravenous catheter was used only once for a second injection in an infant and was not used during the third round of injections, consistent with evidence of a learning curve with the DVI technique in infants and young children.

Pain from DVI was assessed in volunteers aged 6–45 years (groups 1a, 1b, 2a, 2b, 3a, and 3b); 157 of 161 injections (97.5%) were associated with mild or no pain (Supplemental Table 5).

### Safety.

**Figure 2.**
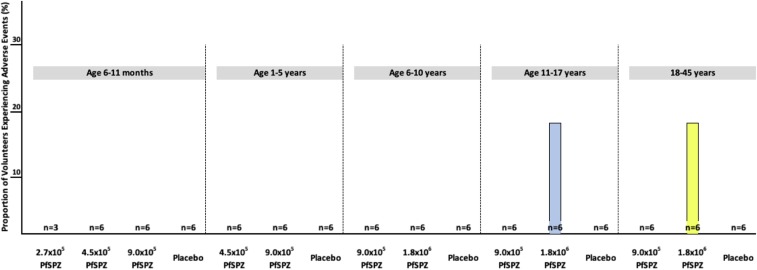
Proportion of volunteers experiencing solicited adverse events (AEs) during the 7 days after each immunization. Ninety-one of 93 volunteers injected at least once experienced no solicited AEs during the 7 days after each immunization. One volunteer experienced three Grade 1 local AEs (yellow bar) and one volunteer experienced Grade 2 temperature elevation (38.5°C) after each immunization accompanied by mild (Grade 1) chills, fatigue, headache, and malaise (blue bar). These AEs are further described in the text. This figure appears in color at www.ajtmh.org.

A global summary of solicited AEs is provided in Table 3. Among the 63 volunteers who received 183 doses of PfSPZ Vaccine, one Group 1b adult volunteer who received 1.8 × 10^6^ PfSPZ reported three solicited local AEs (tenderness and pain after first dose and tenderness after second dose); all were Grade 1 in severity and resolved within 2 days. No solicited local AEs were reported among the 30 volunteers after receiving 90 doses of NS. Solicited systemic AEs were detected after 3/183 injections of PfSPZ and 0/90 injections of NS. All 16 AEs occurred in a single Group 2b volunteer (age 11 years) who received a dose of 1.8 × 10^6^ PfSPZ. All AEs were Grade 1 except elevated temperatures, which were Grade 2. Symptoms included chills and feverishness after two of the three injections with fatigue, headache, malaise, and elevated temperature of 38.5°C after all three injections. The AEs occurred 12–24 hours after each immunization and resolved within 24 hours. This individual had no change in total white blood cell or absolute neutrophil counts or biochemistry values 2 days after each immunization but did have a mild decline in total lymphocyte counts that did not go below the lower limit of normal on Day 2 after each immunization. No other vaccine recipient experienced a systemic solicited AE. The local AEs in the one adult and the systemic AEs in the one adolescent are delineated in Figure 2. No significant differences were found between vaccinees and placebo recipients with respect to systemic or local event rates whether assessed as overall rates or specific rates for each type of AE (*P* = 0.60 for all by Barnard’s test).

**Table 3:** Global adverse event (AE) summary

	Vaccine (*N* = 63)		Placebo (*N* = 30)	
	All AEs	Possibly, probably, or definitely related AEs	All AEs	Possibly, probably, or definitely related AEs
No. of volunteers with at least one solicited AE within 7 days of immunization (%)	2 (3.2%)	2 (3.2%)	0 (0.0%)	0 (0.0%)
Total no. of solicited AEs (maximum severity grade)	19 (Grade 2)*	18 (Grade 2)*	0 (NA)	0 (NA)
No. of volunteers with a solicited Grade 3 AE (%)	0 (0.0%)	0 (0.0%)	0 (0.0%)	0 (0.0%)
No. of volunteers with at least one solicited local AE	1 (1.6%)	1 (1.6%)	0 (0.0%)	0 (0.0%)
Total no. of local AEs (maximum severity grade)	3 (Grade 1)	3 (Grade 1)	0 (NA)	0 (NA)
No. of volunteers with at least one solicited systemic AE (%)	1 (1.6%)	1 (1.6%)	0 (0.0%)	0 (0.0%)
Total no. of systemic AEs (maximum severity grade)	16† (Grade 2)*	15† (Grade 2)*	0 (NA)	0 (NA)
No. of volunteers with at least one unsolicited AE within 28 days of immunization (%)	23 (36.5%)	0 (0.0%)	10 (33.3%)	1 (3.3%)
Total no. of unsolicited AEs within 28 days of immunization (maximum severity grade)	34 (Grade 3)	0 (NA)	11 (Grade 1)	2 (Grade 1)
No. of volunteers with an unsolicited Grade 3 AE (%)	1 (1.6%)	0 (0.0%)	0 (0.0%)	0 (0.0%)
No. of volunteers experiencing an SAE (%)	1 (1.6%)	0 (0.0%)	0 (0.0%)	0 (0.0%)
Total no. of SAEs (maximum severity grade)	1 (Grade 3)	0 (NA)	0 (NA)	0 (NA)

SAE = serious adverse event.

* The only Grade 2 AE was elevated temperature.

† All solicited systemic AEs occurred in a single individual.

Twenty of 63 vaccinees (31.7%) experienced 30 unsolicited AEs (0.48/individual) during the 28 days after each immunization (Table 3). All unsolicited AEs were identified as unlikely related to administration of IP. Three unsolicited AEs were moderate (Grade 2) and one (the serious adverse event [SAE] described subsequently) severe (Grade 3) in severity; all others were mild (Grade 1). Ten of 30 controls (33.3%) experienced 13 unsolicited mild (Grade 1) AEs (0.43/individual) during this period. Two episodes of fever in one volunteer (Group 4a, age 3 years receiving 4.5 × 10^5^ PfSPZ), occurring 14 and 19 days after the first dose of NS, were determined to be possibly related to study product during the blinded safety assessment. Details of the unsolicited AEs can be found in Supplemental Table 6.

One SAE was reported in a 2-year-old volunteer (Group 4a) receiving 4.5 × 10^5^ PfSPZ Vaccine who was hospitalized with multiple injuries after she was struck by a motorcycle. She subsequently recovered and completed participation in the study.

Four volunteers developed parasitemia during the immunization period. Three infections in adults were detected retrospectively by qPCR (Supplemental Table 7); these individuals were TBS negative throughout this period of the study. Two of the three cases were Pm infections and one case was Pf, the latter confirmed by genotyping to differ from the NF54 strain of Pf used in the vaccine (Supplemental Table 8). The two individuals with Pm were determined retrospectively to be positive by qPCR at the time of first immunization and remained positive during the entire 16-week immunization period until treated after the third immunization. The individual with Pf infection was negative at the time of the first immunization, developed Pf infection before the second immunization, and remained positive until treated after the third immunization. All three volunteers were treated with artesunate–amodiaquine once the polymerase chain reaction results were known. A fourth volunteer, from Group 5 (ages 6–12 months), who received NS, was positive by qPCR for Pf genotypically distinct from the NF54 vaccine strain (Supplemental Tables 7 and 8) before the first and second immunizations; parasitemia in this infant was detected in real time by TBS before the second immunization, leading to immediate treatment with artemether–lumefantrine (Supplemental Table 7). No signs of illness were reported by the mother for this infant, who continued in the trial.

No clinically significant laboratory abnormalities were attributed to PfSPZ (Supplemental Table 9). The most commonly identified abnormalities listed in order of prevalence included anemia, lymphopenia, and leukopenia, with no difference in frequency across age groups or between vaccine recipients and NS controls in each age group. Two volunteers experienced a Grade 3 laboratory abnormality: an isolated occurrence of lymphopenia in a Group 1b adult volunteer 28 days after the first dose of 1.8 × 10^6^ PfSPZ in association with a concomitant viral infection, and neutropenia in a Group 5b volunteer receiving 4.5 × 10^5^ PfSPZ determined to have benign ethnic neutropenia. Both abnormalities resolved without sequelae.

### Antibody responses to Pf.

**Figure 3.**
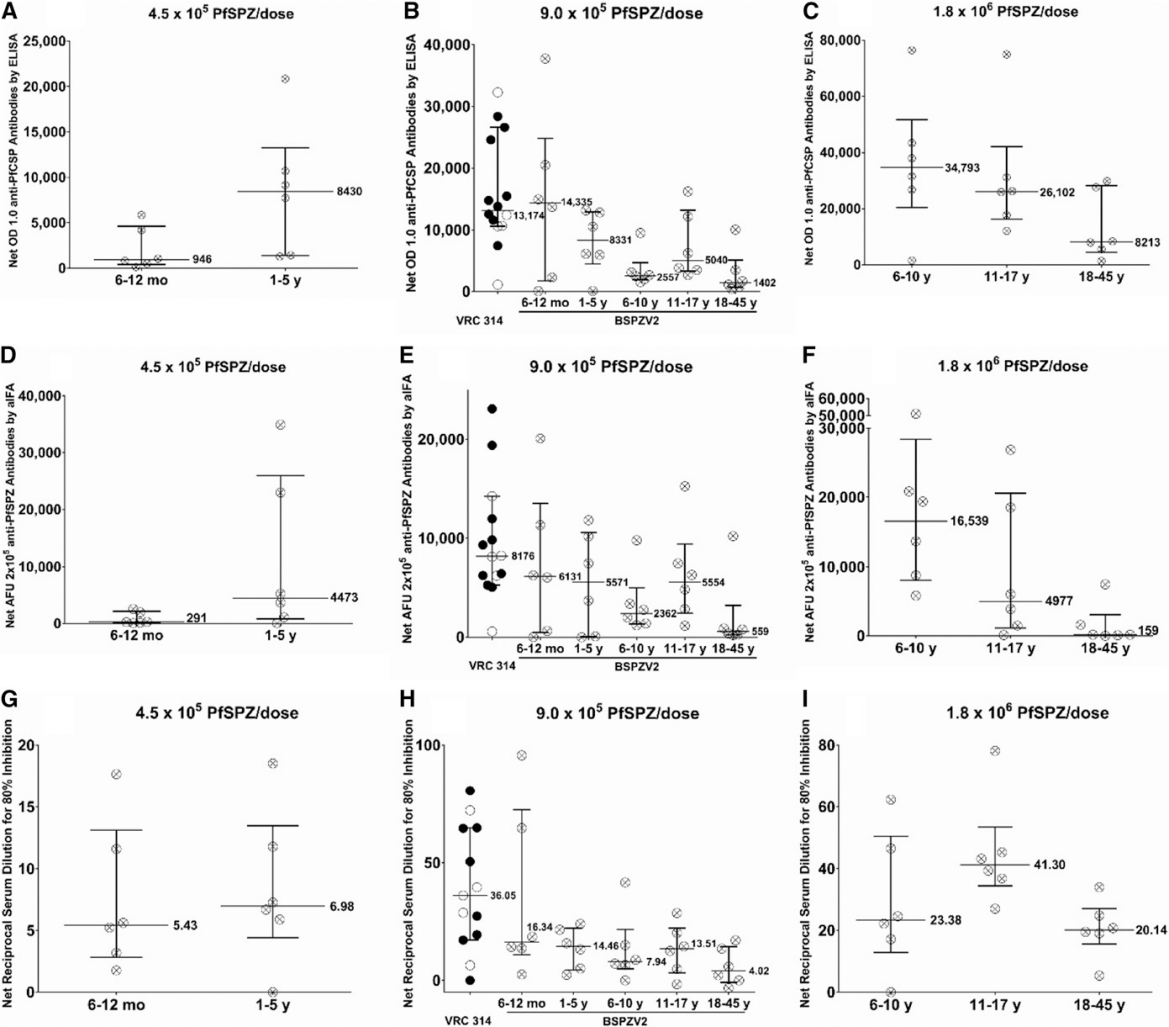
Difference between postimmunization and preimmunization antibody results. Antibody assay results by dose in *Plasmodium falciparum* circumsporozoite protein enzyme-linked immunosorbent assay (**A**–**C**), immunofluorescence assay (**D**–**F**), and inhibition of sporozoite invasion (**G**–**I**) assays. Results were obtained by subtracting preimmune values from the values obtained from sera drawn 2 weeks after the third dose. For the 9.0 × 10^5^ *Plasmodium falciparum* sporozoite dose (**B**, **E**, and **H**), previously assessed results from clinical trial VRC 314,^17^ conducted in the United States with the same dosage regimen and in the same laboratory with the same assays as in the BSPZV2 trial assays, are shown as a comparison. For the VRC 314 trial^17^ the filled in circles indicate protected volunteers and the empty circles the unprotected volunteers. Medians with interquartile ranges are shown.

Antibodies against PfCSP by ELISA, PfSPZ by aIFA, and PfSPZ by aISI in sera taken before immunization and 2 weeks after last vaccine dose are shown in Supplemental Table 10, Figure 3, and Supplemental Figure 1 for vaccinees and Supplemental Table 11 for controls.

In the PfCSP ELISA, volunteers were considered to have made a positive response if their net optical density (OD) 1.0 (Figure 3A–C) and OD 1.0 ratio (Supplemental Figure 1A–C), calculated, respectively, by subtracting or dividing by the prevaccination antibody OD 1.0, were ≥ 50 and ≥ 3.0, respectively. By these criteria, 59/60 vaccinees developed antibodies to PfCSP. The only volunteer who did not have a positive response to immunization was a 20-year old who received the 9.0 × 10^5^ PfSPZ regimen (Supplemental Table 10). The overall highest responses were in 6–10-year olds who received 1.8 × 10^6^ PfSPZ and had a median net OD 1.0 of 34,793 and median net OD 1.0 ratio of 15,515 (Supplemental Table 8). Only 2/30 of the NS controls, both infants, developed antibodies to PfCSP (Supplemental Table 11).

In the aIFA, volunteers with a net arbitrary fluorescence unit (AFU) 2 × 10^5^ of ≥ 150 (Figure 3D–F) and a ratio of post- to pre-AFU 2 × 10^5^ of ≥ 3.0 (Supplemental Figure 1, panels D-F) were considered to have made a positive response. By these criteria, 57/60 volunteers made a positive response to immunization. The three volunteers who did not make a positive response were 1 year, 7 months, and 9 months of age at the time of first injection and received the 9.0 × 10^5^, 4.5 × 10^5^, and 9.0 × 10^5^ PfSPZ dosing regimens, respectively (Supplemental Table 10). As with the PfCSP ELISA, the overall highest responses were in 6–10-year olds who received the 1.8 × 10^6^ PfSPZ regimen and had a median net AFU 2 × 10^5^ of 20,099 and median net AFU 2 × 10^5^ ratio of 16,539 (Supplemental Table 10). None of the NS controls developed antibodies to PfSPZ by aIFA (Supplemental Table 11).

In the automated inhibition of sporozoite invasion assay (ISI), volunteers with a net ISI activity of ≥ 10% (Figure 3G–I) and ratio of post to pre-ISI activity of ≥ 3.0 (Supplemental Figure 1, panels G-I) were considered positive. By these criteria, 37/60 volunteers had a positive response to immunization. The only group with a 100% response rate (6/6) was the 11–15-year olds who received the 1.8 × 10^6^ PfSPZ regimen. This group also had the highest median net 80% ISI activity (41.3) and median net 80% ISI activity ratio (26.8). Only 2/30 of the NS controls developed antibodies to PfSPZ by aISI, a 38 and a 13-year old (Supplemental Table 11).

Adults (18–35 years), teenagers (11–15 years), and older children (6–10 years) received the 1.8 × 10^6^ PfSPZ dosage regimen, and these age groups and the younger children (1–5 years) and infants (7–11 months) received the 9.0 × 10^5^ PfSPZ regimen; the younger children and infants also received the 4.5 × 10^5^ the PfSPZ regimen. We, therefore, assessed the effect of age on immunogenicity. This is shown graphically using net values in Figure 3 and for ratios in Supplemental Figure 1 and in Supplemental Tables 10 and 11. For the 1.8 × 10^6^ PfSPZ dosage regimen, the adults had the lowest antibody responses by all assays. The older children had the highest responses in the PfCSP ELISA and aIFA and teenagers for the aISI. For the 9.0 × 10^5^ PfSPZ dosage regimen, the adults had the lowest median net OD 1.0, median net AFU 2 × 10^5^, and median net 80% ISI responses, and the lowest ratios for PfCSP ELISA and aISI; infants had the highest responses for all of these assays. For the median AFU 2 × 10^5^ ratio, the teenagers had the best response. For the 9.0 × 10^5^ PfSPZ dosage regimen, the median OD 1.0 and median OD 1.0 ratios in the PfSPZ ELISA were 10.2 and 165.7 times higher in the infants as compared with the adults. For the aIFA, they were 11.0 and 3.3 times higher and for aISI, they were 4.1 and 6.3 times higher, respectively (Figure 3, Supplemental Figure 1, Supplemental Table 10). Because of the small sample sizes (*N* = 6) and variability within each group, the differences did not quite reach the level of statistical significance.

Adults, teenagers, and older children received the 1.8 × 10^6^ or 9.0 × 10^5^ regimens. For all three assays, the response to the 1.8 × 10^6^ regimen was higher than the response to the 9.0 × 10^5^ regimen (Figure 3, Supplemental Figure 1, Supplemental Table 10). Younger children and infants received the 9.0 × 10^5^ and 4.5 × 10^5^ regimens. For all three assays, the response to the 9.0 × 10^5^ PfSPZ regimen was higher than the response to the 4.5 × 10^5^ regimen in infants. However, for the younger children, this was not the case (Figure 3, Supplemental Figure 1, Supplemental Table 10).

In previous clinical trials with PfSPZ Vaccine in adults in the United States with no previous exposure to malaria, there has been a significant correlation between the three different antibody assays^14–17^ in sera taken 2 weeks after the last dose of vaccine. In this clinical trial, we assessed the correlation between the assays for sera taken 2 weeks after the third (last) dose of vaccine from 60 volunteers. There was a significant correlation between the results of the PfCSP ELISA and the aISI (*R*^2^ = 0.45, *P* < 0.0001). The correlations between PfCSP ELISA and aIFA (*R*^2^ = 0.05, *P* = 0.085) and aIFA versus aISI (*R*^2^ = 0.01, *P* = 0.40) were not significant.

Having demonstrated that median antibody responses in Tanzanian infants were consistently higher than in Tanzanian adults, we compared the Tanzanian responses to those in adults in the United States who received three doses of 9.0 × 10^5^ PfSPZ (Figure 3B, E, and H). Median antibody responses in U.S. adults and Tanzanian infants were, respectively: 1) 13,174 and 14,335 for PfCSP ELISA, 2) 8,176 and 6,131 for aIFA, and 3) 36.05 and 16.34 for aISI.

### T-cell responses to PfSPZ.

**Figure 4.**
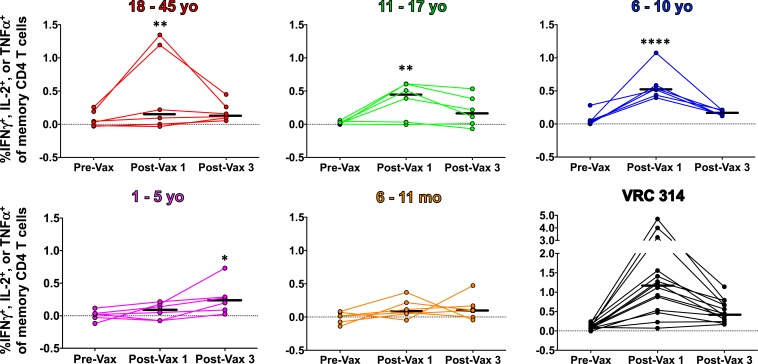
*Plasmodium falciparum* sporozoites (PfSPZ)-specific memory CD4 T-cell responses pre- and postvaccination. Percent of memory CD4 T cells in the blood expressing interferon gamma (IFN-γ) interleukin 2 (IL-2) or tumor necrosis factor alpha (TNF-α) at preimmunization or 2 weeks after the first and third doses of PfSPZ Vaccine (9.0 × 10^5^). Results are the percentage of cytokine-producing cells after incubation with PfSPZ minus the percentage of cytokine-producing cells after incubation with vaccine diluent (medium with 1% human serum albumin). Bars indicate median values within each group. Differences within each age group between pre- and postvaccination groups were assessed by two-way ANOVA with Dunnett’s correction for multiple comparisons. *P < 0.05, **P < 0.01, ****P < 0.0001. Previously measured results from clinical trial VRC 314^17^ conducted in the United States with the same dosage regimen and the same assay conducted in the same laboratory as for the BSPZV2 trial assays are shown as a comparison. This figure appears in color at www.ajtmh.org.

T-cell responses to PfSPZ were assessed by polychromatic flow cytometry on cryopreserved PBMCs acquired before immunization, 2 weeks after the first dose of PfSPZ Vaccine and 2 weeks after the third dose of vaccine in subjects who received three doses of 9.0 × 10^5^ PfSPZ of PfSPZ Vaccine (Figure 4 and Supplemental Table 12). Following the first dose of PfSPZ Vaccine, 18–45-year olds, 11–17-year olds, and 6–10-year olds all had significant increases in the frequency of cytokine-producing memory CD4 T cells compared with the prevaccination time point. However, only the 6–10-year-old group had a 100% response rate to the vaccine. In addition, the responses in the 6–10-year-old group were significantly higher than those in the 1–5-year-old and 6–11-month-old groups, but not significantly different than the responses in the other groups (as assessed by the Kruskal–Wallis test with Dunn’s correction for multiple comparisons). Following the third vaccination, only the 1–5-year olds had a significant increase over the prevaccination time point. Infants did not have a significant increase in CD4 T-cell responses at any time point. At no time point were PfSPZ specific memory CD8 T-cell responses detected over background in any group.

## DISCUSSION

Before this study, aseptic, purified, cryopreserved PfSPZ-based products (Sanaria PfSPZ Vaccine, PfSPZ Challenge, and PfSPZ-CVac) had only been injected into adults.^13–19,27,29,35–41^ This was the first study to assess the safety, tolerability, feasibility, and immunogenicity of any PfSPZ-based product in adolescents, children, or infants.

Because no licensed vaccine against an infectious agent is administered by DVI, there was initially a concern in the vaccinology community about the safety, tolerability, and feasibility of administering PfSPZ-based products by DVI to adults. Clinical trials in the United States, Germany, Spain, Mali, Tanzania, and Gabon established that rapid administration by DVI of PfSPZ products in 0.5 mL of diluent through a 25-gauge needle was safe, extremely well tolerated, straightforward, and protective.^16,18,27,38,39,41^ After establishing the safety, tolerability, and feasibility of PfSPZ administration by DVI in adults, there was still concern that DVI administration of PfSPZ might be problematic in younger age groups as no preventative vaccine has ever been administered by intravenous injection (IV)/DVI to these age groups. This concern was not borne out by the findings of the present study.

There were no significant differences in solicited AEs between vaccinees in any age group (*N* = 63) and corresponding controls (*N* = 30) who received NS (*P* = 0.6). Furthermore, there were no differences in AEs between different dosage regimens, no differences between age groups, and no differences between the first, second, and third immunizations. PfSPZ Vaccine was extremely well tolerated. We have no explanation for the symptoms and elevated temperature experienced by the 11-year-old girl after all three injections, which resolved within 24 hours of each immunization. This has not occurred in any subject in any other clinical trial of PfSPZ Vaccine; we will monitor for this pattern in future trials.

Moreover, the administration process itself (DVI) was extremely well tolerated. Six- to 45-year olds were questioned about pain after each injection. Direct venous inoculation was associated with mild or no pain for 157 of 161 injections (97.5%); 129 of 161 injections (80.1%) were associated with no pain (Supplemental Table 5).

There was no significant difference in the feasibility of administration of PfSPZ (or NS placebo) to adults, adolescents, 6–10-year olds, or 1–5-year olds. Administration was achieved with the first needle stick in 53/54 (98%) administrations in 18–45-year olds, 51/54 (94%) administrations in 11–17-year olds, 47/54 (87%) administrations in 6–10-year olds, and 47/54 (87%) administrations in 1–5-year olds (Supplemental Table 5). Administration success with one needle stick decreased to 35/57 (61%) injections in infants (Supplemental Table 5). However, in infants, the learning curve of the nurses administering the vaccine was rapid. In infants, DVI was successful on the first attempt in nine of 21 (43%) for first dose, 11 of 18 (61%) for second dose, and 15 of 18 (83%) for the third dose. The option to establish intravenous access with an intravascular catheter was used for 12 first injections (nine infants), but only once for a second injection (one infant) and was not used during the third round of immunizations, consistent with evidence of a learning curve with the technique when administering to infants.

The levels of antibodies to PfCSP by ELISA were 31 times lower in adults in Mali than in U.S. adults and 4.3 times lower in adults in Tanzania,^20^ who received the exact same immunization regimen.^18^ We hypothesized that this was due to immunoregulation after long-term exposure to Pf infections and that in malaria-endemic areas, antibody responses would be higher in children and infants who had less exposure to Pf than in adults with long-term exposure. Naturally acquired immunity may also have affected the viability of the sporozoites, and this also is most highly developed in adults with long-term exposure. However, the levels of antibody preimmunization and in the placebo controls were quite low, arguing against this explanation for reduced immunogenicity (Supplemental Tables 10 and 11). Results from this study are consistent with the hypothesis that reduced immune responses in semi-immune African as compared with nonimmune American adults was due to immunoregulation after long-term exposure to Pf infections, and that in malaria-endemic areas antibody responses would be higher in children and infants who had less exposure to Pf than in adults with long-term exposure. The median antibody responses by PfCSP ELISA, PSPZ aIFA, and PfSPZ aISI were highest in infants and lowest in adults (Figure 3), and the antibody responses in infants were comparable with the antibody responses seen in adults in the United States who received the identical immunization regimen (Figure 3). However, differences among age groups did not quite reach the level of statistical significance because of the small sample size and the variance. Ongoing studies will establish whether this age effect is consistent and significant.

T-cell responses against PfSPZ have been demonstrated in malaria-naïve adults immunized with PfSPZ Vaccine in the United States^14,16^ and in a previous study of PfSPZ Vaccine in adults in Tanzania,^20^ but they were much lower in Tanzania than in the United States after administration of the same immunization regimen. In this trial, there were no CD8 T-cell responses against PfSPZ detected. However, significant increases in peripheral CD4 T-cell responses were seen in all age groups except infants after in vitro stimulation with PfSPZ (Supplemental Table 12). The median adult responses were about six times lower than they were after immunization with the same regimen in the United States (Figure 4).^17^ Consistent with all of our trials, the best responses in adults, 11–17-year olds, and 6–10-year olds were seen after the first dose of PfSPZ Vaccine^14–17^ with the highest responses in 6–10-year olds. We have previously hypothesized that the reason peak T-cell responses are highest after the first dose is that after the first dose the functionally important T cells are resident in the liver and, thus, are not detected in the periphery.^14^ However, after the third dose of PfSPZ Vaccine, the only significant results were in 1–5-year olds; this was the best response recorded for this age group. Perhaps, because of immunological immaturity, it took longer to prime 1–5-year olds, and if they were administered more priming doses earlier, they would have better responses. Infants did not have any evidence of induction of T-cell responses. Such poor T-cell responsiveness in infants has also been observed following hepatitis B and oral polio vaccinations.^42,43^ This is likely based on the fact that T-cell repertoires in neonates and infants are skewed toward Th2-type responses.^44–48^ Since PfSPZ Vaccine is thought to rely primarily on T-cell responses to mediate protection,^14,15,17,49^ the T-cell studies may indicate that children, but not infants, will be protected by the immunization regimen (three doses at 8-week intervals) used in this study. We are now exploring priming regimens in which multiple doses of PfSPZ are administered during the first week,^27^ and this may prove to be a more powerful method of priming and could thereby overcome the poor T-cell responsiveness we have identified in infants. However, we recognize that we will be breaking new ground here, as to our knowledge, there are no data in infants for any vaccine that this can be done. In addition, we will explore the impact of booster doses during the first or second years of life, and we are developing an adjuvant that may be useful.

By establishing the safety, tolerability, and feasibility of administration of PfSPZ Vaccine to children and infants, this trial was an important prelude to clinical trials in more than 300 infants being conducted in Kenya (ClinicalTrials.gov NCT02687373) and Equatorial Guinea (ClinicalTrials.gov NCT02859350). It also supported our long-term plans to use PfSPZ Vaccine in mass vaccination programs (MVPs) to focally eliminate malaria. Because high population coverage will be needed to halt transmission, any vaccine intended for this purpose must be extremely safe, easy to administer, and minimally painful for the vaccinee, in all age groups. As there was no difference in the rate of AEs or laboratory abnormalities between any vaccine group and placebo recipients and because 97.2% of volunteers old enough to evaluate pain experienced no pain or only mild pain during administration, PfSPZ Vaccine appeared in the present study to be highly suitable for use in MVPs.

The results demonstrated that 6–10-year olds and 1–5-year olds have the highest CD4 T-cell responses after the first and third doses of PfSPZ Vaccine, respectively. These findings raise hope that the vaccine will be more protective in children than it was in adults in Mali.^18^ This will soon be assessed in 1–12-year olds in Gabon. However, because no T-cell responses were identified in infants, we are concerned about infants’ immunological capacity to mount protective T-cell responses after this immunization regimen of PfSPZ Vaccine. This is now being assessed in the Kenya study, and if it holds up, we may have to alter the immunization regimen (number of PfSPZ per dose, number of doses, and interval between doses). If this is not successful, we would likely initiate immunization only after the first year of life. PfSPZ Vaccine is intended to be used in MVPs to halt transmission of Pf and not in a routine infant immunization program (Expanded Program for Immunization). In such MVPs, we would cover the infants with antimalarial drugs until they reached 1–2 years of age when immunizations with PfSPZ Vaccine would begin.

## Supplementary Files

Supplemental figures and tables
