# Supplementary material for: Safety and Differential Antibody and T-Cell Responses to the Plasmodium falciparum Sporozoite Malaria Vaccine, PfSPZ Vaccine, by Age in Tanzanian Adults, Adolescents, Children, and Infants
Source: Am J Trop Med Hyg. 2019 Apr 15;100(6):1433–44. doi: 10.4269/ajtmh.18-0835 (PMC6553883; doi:10.4269/ajtmh.18-0835)
Supplement: Supplementary file 1 [file tpmd180835.SD1.pdf]

Supplementary Data

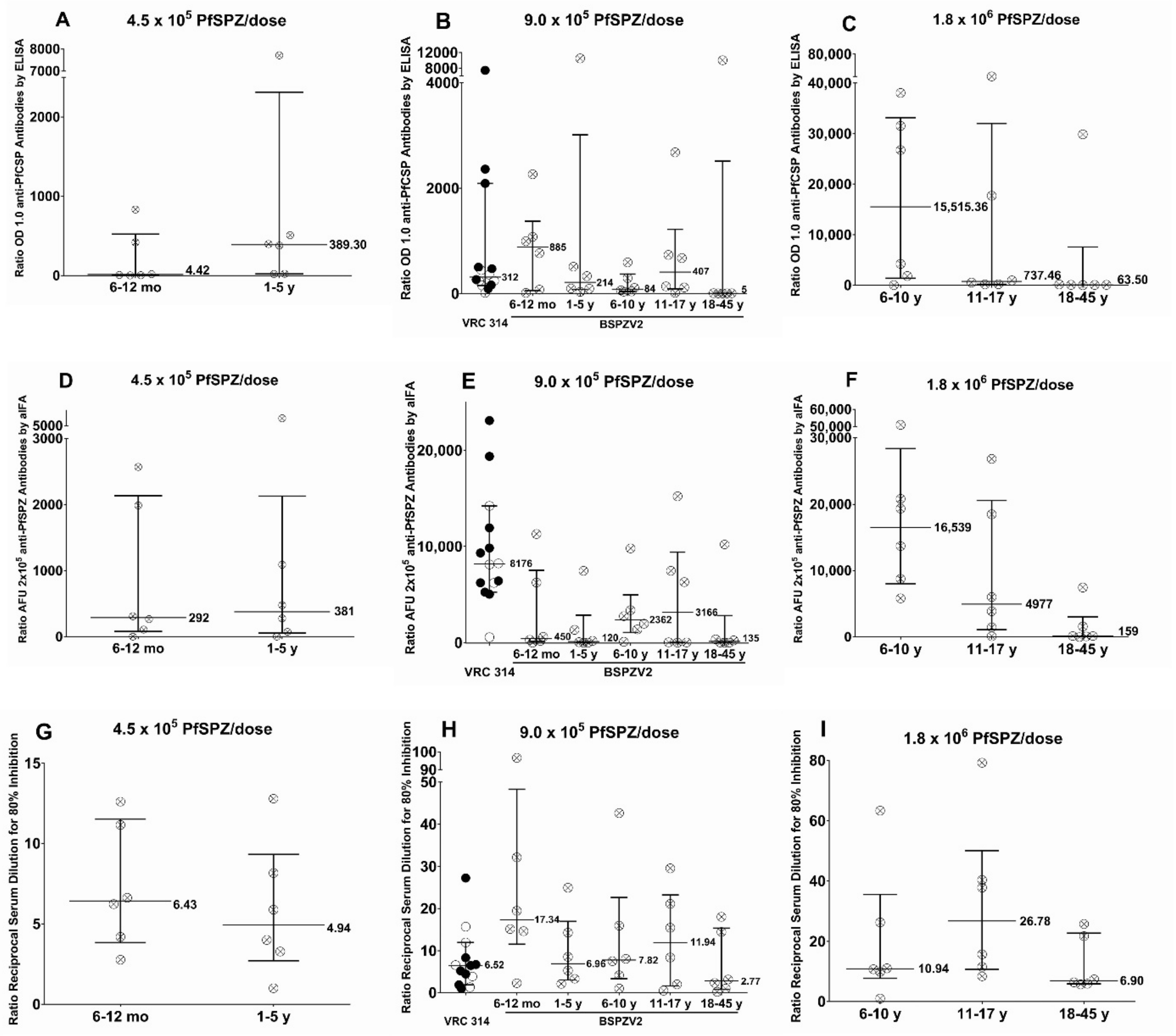

Fig. S1. Ratio of post-immunization to pre-immunization antibody results. Antibody assay results by dose in PfCSP ELISA (A-C), aIFA (D-F), and aISI (G-I) assays, are expressed as ratios. Results were obtained by dividing values obtained from sera drawn 2 weeks after the last dose of vaccine by values obtained from sera drawn pre-immunization. For the 9.0 x 10^5 PfSPZ dose (B, E, H), previously measured results from clinical trial VRC 314[17], conducted in the United States with the same dosage regimen, are shown as a comparison. For the VRC 314[17] trial

the filled-in circles indicate protected volunteers and the empty circles the unprotected volunteers. Medians with interquartile ranges are shown.

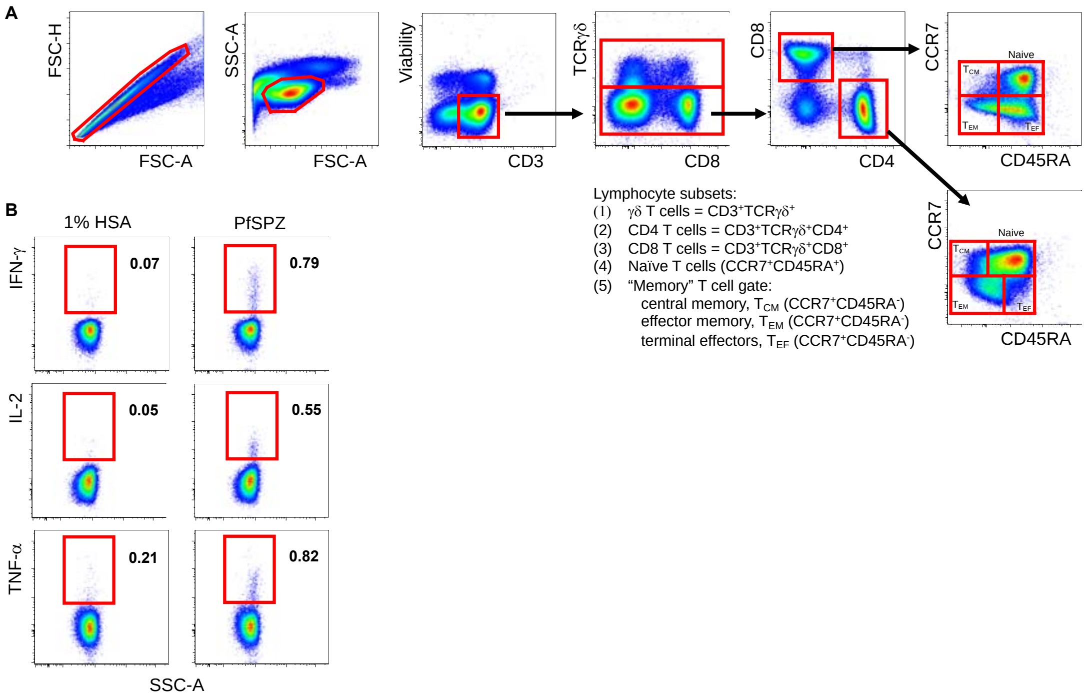

Fig. S2. Flow cytometry gating for analysis of human PBMCs. (A) Gating tree for analysis of memory CD4 and CD8 T cells. (B) Frequencies of cytokine-producing memory CD4 T cells following stimulation with either PfSPZ or vaccine diluent (1% HSA). PfSPZ-specific memory CD4 T cells are identified by expression of any combination of IFN-γ, IL-2, or TNF-α.

Table S1. Test completed successfully by volunteers before included in study.

All questions are answered true or false.

1. The main purpose of the study is to evaluate the safety, tolerability, immunogenicity, and protective efficacy against CHMI (adults only) of PfSPZ Vaccine
2. Volunteers have the right to discontinue their participation at any time without asking for permission
3. Risks for volunteers are related to exposure to PfSPZ Vaccine and needle prick during blood sampling
4. The duration of participation in the study may differ from one group to another group.
5. The study vaccine will be given intravenously
6. Volunteers must inform a study doctor of any medical problem they may have
7. It is not necessary for a volunteer to provide his/her consent before any study procedures performed on him/her
8. Volunteering in this study is one way of earning money
9. Some of volunteers' blood samples will be stored and may be used by researchers at a later time
10. Volunteers who will be tested positive for HIV and/or hepatitis will be counseled and referred to appropriate clinic for further management

Table S2. Inclusion criteria.

1. Healthy males and females, based on clinical and laboratory findings
2. From the age 6 months to 45 years
3. Adults with a Body Mass Index (BMI) 18 to 30 Kg/m^2; or adolescents, children and infants with Z-score of the selected indicator ([weight-for-height], [(height and BMI) for age]) category within ±2SD
4. Long term (at least one year) or permanent residence in the Bagamoyo town or nearby villages
5. Agreement to release medical information and to inform the study doctor concerning contraindications for participation in the study
6. Willingness to be attended to by a study clinician and take all necessary medications prescribed during study period
7. Agreement to provide contact information of a third party household member or close friend to study team
8. Availability through mobile phone 24 hours during the entire study period
9. Agreement not to participate in another clinical trial during the study period
10. Agreement not to donate blood during the study period
11. Able and willing to complete the study visit schedule over the study follow up period, including the hospitalizations required for protocol compliance
12. Willingness to undergo HIV, hepatitis B (HBV) and hepatitis C (HCV) tests
13. Volunteer (subjects 18 years of age and older) and parent or guardian signing informed consent (for subjects <18 years of age) is able to demonstrate their understanding of the study

by responding correctly to 10 out of 10 true/false statements (in a maximum of two attempts for those who failed to respond correctly to all true/false statements in the first attempt)

14. Signed written informed consent, in accordance with local practice, provided by adult volunteers, parents or legal representatives and relevant assent for children participants as applicable
15. Free from malaria parasitaemia by blood smear at enrolment
16. Free from helminth infections at enrolment, or diagnosed with helminths and treated appropriately to eliminate infestation
17. Female volunteers aged 9 years and above must be non-pregnant (as demonstrated by a negative serum pregnancy test), and provide consent / assent of their willingness to take protocol-defined measures not to become pregnant during the study and safety follow-up period

Table S3. Exclusion criteria.

1. Previous receipt of an investigational malaria vaccine or drug in the last 5 years
2. Participation in any other clinical study involving investigational medicinal products within 30 days prior to the onset of the study or during the study period
3. History of arrhythmias or prolonged QT-interval or other cardiac disease, or Clinically significant abnormalities in electrocardiogram (ECG) at screening
4. Positive family history in a 1st or 2nd degree relative for cardiac disease at age <50 years old
5. A history of psychiatric disease
6. Suffering from any chronic illness including; diabetes mellitus, cancer or HIV/AIDS
7. Any confirmed or suspected immunosuppressive or immune-deficient condition, including asplenia
8. History of drug or alcohol abuse interfering with normal social function
9. The use of chronic immunosuppressive drugs or other immune modifying drugs within three months of study onset (inhaled and topical corticosteroids are allowed) and during the study period
10. Any clinically significant deviation from the normal range in biochemistry or hematology blood tests or in urine analysis
11. Positive HIV, hepatitis B virus or hepatitis C virus tests
12. Volunteers who are suspected as having clinically active TB by history or physical examination with positive QuantiFERON-TB Gold Test In-Tube assay
13. Symptoms, physical signs and laboratory values suggestive of systemic disorders including renal, hepatic, blood, cardiovascular, pulmonary, skin, immunodeficiency, psychiatric, and

other conditions which could interfere with the interpretation of the study results or compromise the health of the volunteers

14. Any medical, social condition, or occupational reason that, in the judgment of the investigator, is a contraindication to protocol participation or impairs the volunteer's ability to give informed consent, increases the risk to the volunteer because of participation in the study, affect the ability of the volunteer to participate in the study or impair interpretation of the study data

Table S4. Solicited adverse events (defined as within 7 days of each immunization).

	Local	Systemic
Groups 1, 2 and 3 (6 to 45 years)	By history:<br>• Pain<br>• Pruritis<br>By visualization:<br>• Bruising/extravasated blood<br>• Erythema<br>• Swelling<br>By palpation:<br>• Tenderness<br>• Induration	By history:<br>• Subjective fever<br>• Headache<br>• Fatigue<br>• Malaise<br>• Chills<br>• Myalgia<br>• Arthralgia<br>By examination:<br>• Temperature ≥ 38°C<br>• Allergic reaction (rash, urticaria, pruritis, edema)
Groups 4 and 5 (6 months to 5 years)	By visualization:<br>• Bruising/extravasated blood<br>• Erythema<br>• Swelling<br>By palpation:<br>• Tenderness<br>• Induration	By history:<br>• Fever<br>• Drowsiness<br>• Irritability/fussiness<br>• Inability/refusal to eat or drink<br>By examination:<br>• Temperature ≥ 38°C

Table S5. Characteristics of DVI

		Group 1 (18-45 years)			Group 2 (11-17 years)			Group 3 (6-10 years)		
		**$9x10^5$ All Injections**	**$1.8x10^6$ All Injections**	**Placebo All Injections**	**$9x10^5$ All Injections**	**$1.8x10^6$ All Injections**	**Placebo All Injections**	**$9x10^5$ All Injections**	**$1.8x10^6$ All Injections**	**Placebo All Injections**
Total number of syringes	1	18 (100.0%)	18 (100.0%)	18 (100.0%)	18 (100.0%)	18 (100.0%)	18 (100.0%)	18 (100.0%)	17 (94.4%)	18 (100.0%)
	2	0 (0%)	0 (0%)	0 (0%)	0 (0%)	0 (0%)	0 (0%)	0 (0%)	1 (5.6%)	0 (0%)
Volunteer pain assessment	Painless	11 (61.1%)	13 (72.2%)	16 (88.9%)	16 (88.9%)	16 (88.9%)	17 (94.4%)	13 (72.2%)	17 (94.4%)	10 (55.6%)
	Mild	6 (33.3%)	5 (27.8%)	2 (11.1%)	2 (11.1%)	2 (11.1%)	0 (0%)	5 (27.8%)	1 (5.6%)	6 (33.3%)
	Moderate	1 (5.6%)	0 (0%)	0 (0%)	0 (0%)	0 (0%)	1 (5.6%)	0 (0%)	0 (0%)	2 (11.1%)
	Severe	0 (0%)	0 (0%)	0 (0%)	0 (0%)	0 (0%)	0 (0%)	0 (0%)	0 (0%)	0 (0%)
Total number of needle sticks	1	17 (94.4%)	18 (100.0%)	18 (100.0%)	17 (94.4%)	16 (88.9%)	18 (100.0%)	16 (88.9%)	16 (88.9%)	15 (83.3%)
	2	1 (5.6%)	0 (0%)	0 (0%)	1 (5.6%)	2 (11.1%)	0 (0%)	2 (11.1%)	1 (5.6%)	3 (16.7%)
	3	0 (0%)	0 (0%)	0 (0%)	0 (0%)	0 (0%)	0 (0%)	0 (0%)	1* (5.6%)	(0%)
	≥ 4	0 (0%)	0 (0%)	0 (0%)	0 (0%)	0 (0%)	0 (0%)	0 (0%)	0 (0%)	0 (0%)
If ONLY 1 stick	Simple	16 (94.1%)	18 (100.0%)	17 (94.4%)	16 (94.1%)	16 (100.0%)	17 (94.4%)	15 (93.8%)	16 (100.0%)	13 (86.7%)

		Group 1 (18-45 years)			Group 2 (11-17 years)			Group 3 (6-10 years)		
		**$9x10^5$ All Injections**	**$1.8x10^6$ All Injections**	**Placebo All Injections**	**$9x10^5$ All Injections**	**$1.8x10^6$ All Injections**	**Placebo All Injections**	**$9x10^5$ All Injections**	**$1.8x10^6$ All Injections**	**Placebo All Injections**
injection was required, how would you rate the procedure?	Moderate Difficulty	1 (5.9%)	0 (0%)	1 (5.6%)	1 (5.9%)	0 (0%)	1 (5.6%)	1 (6.3%)	0 (0%)	2 (13.3%)
	Very Difficult	0 (0%)	0 (0%)	0 (0%)	0 (0%)	0 (0%)	0 (0%)	0 (0%)	0 (0%)	0 (0%)
Minutes between thawing to syringe hand-off	n	18	18	18	18	18	18	18	18	18
	Mean (SD)	9.6 (0.9)	7.3 (1.9)	7.9 (2.3)	8.8 (1.1)	7.7 (2.4)	7.5 (1.4)	8.7 (1.6)	8.2 (2.3)	7.1 (0.8)
	(Min, Max)	(8,11)	(5,13)	(5,13)	(8,11)	(6,17)	(4,11)	(6,13)	(7,17)	(6,9)
Minutes between syringe hand-off to injection completion	n	18	18	18	18	18	18	18	18	18
	Mean (SD)	2.1 (1.2)	2.1 (0.9)	1.8 (0.6)	2.5 (1.3)	2.8 (3.2)	2.2 (1.1)	2.6 (1.6)	1.7 (1.0)	2.5 (1.7)
	(Min, Max)	(1,5)	(1,4)	(1,3)	(1,6)	(1,15)	(1,5)	(1,8)	(1,4)	(1,7)

		Group 4 (1-5 years)			Group 5 (6-11 months)			
		**4.5x10^5 All Injections**	**9x10^5 All Injections**	**Placebo All Injections**	**2.7x10^5 All Injections**	**4.5x10^5 All Injections**	**9x10^5 All Injections**	**Placebo All Injections**
Total number of syringes	1	18 (100.0%)	18 (100.0%)	18 (100.0%)	1 (33.3%)	15 (83.3%)	16 (88.9%)	18 (100.0%)
	2	0 (0%)	0 (0%)	0 (0%)	2 (66.7%)	3 (16.7%)	2 (11.1%)	0 (0%)
Did the child cry?	No	8 (44.4%)	6 (33.3%)	7 (38.9%)	0 (0%)	3 (16.7%)	1 (5.6%)	2 (11.1%)
	Yes	10 (55.6%)	12 (66.7%)	11 (61.1%)	3 (100.0%)	15 (83.3%)	17 (94.4%)	16 (88.9%)
Total number of needle sticks	1	16 (88.9%)	14 (77.8%)	17 (94.4%)	1 (33.3%)	9 (50.0%)	11* (61.1%)	15 (83.3%)
	2	2 (11.1%)	3* (16.7%)	1 (5.6%)	0 (0%)	3 (16.7%)	3** (16.7%)	3 (16.7%)
	3	0 (0%)	1* (5.6%)	0 (0%)	1* (33.3%)	3** (16.7%)	2* (11.1%)	0 (0%)
	≥ 4	0 (0%)	0 (0%)	0 (0%)	1* (33.3%)	3* (16.7%)	2* (11.1%)	0 (0%)
If ONLY 1 stick injection was required, how would you rate the procedure?	Simple	16 (100.0%)	13 (92.9%)	17 (100.0%)	1 (100.0%)	9 (100.0%)	11 (100.0%)	15 (100.0%)
	Moderately Difficult	0 (0%)	1 (7.1%)	0 (0%)	0 (0%)	0 (0%)	0 (0%)	0 (0%)
	Very Difficult	0 (0%)	0 (0%)	0 (0%)	0 (0%)	0 (0%)	0 (0%)	0 (0%)
Minutes between	n	18	18	18	3	18	18	18

		Group 4 (1-5 years)			Group 5 (6-11 months)			
		**$4.5x10^5$ All Injections**	**$9x10^5$ All Injections**	**Placebo All Injections**	**$2.7x10^5$ All Injections**	**$4.5x10^5$ All Injections**	**$9x10^5$ All Injections**	**Placebo All Injections**
Thawing to Syringe hand-off	Mean (SD)	5.8 (1.1)	6.9 (2.0)	6.6 (1.4)	6.3 (1.5)	6.6 (2.6)	6.7 (2.0)	6.4 (0.9)
	(Min, Max)	(4,8)	(4,10)	(4,9)	(5,8)	(4,16)	(4,9)	(5,8)
Minutes between Syringe hand-off to Injection Completion	n	18	18	18	3	18	18	18
	Mean (SD)	2.5 (1.6)	3.7 (3.7)	2.3 (0.9)	2.0 (1.0)	5.7 (5.4)	3.8 (3.9)	5.1 (4.8)
	(Min, Max)	(1,8)	(1,17)	(1,4)	(1,3)	(2,20)	(1,14)	(2,21)

* - number includes insertion of an intravenous catheter as the final needle stick for 1 volunteer

** - number includes insertion of an intravenous catheter as the final needle stick for 2 volunteers

Table S6. Unsolicited AEs by volunteer

Group	Dose	Volunteer ID	Adverse Event	Associated with Dose #	# of Days Post Associated Dose	Duration (days)	Severity	Relationship to Study Product	Outcome
1a	$9x10^5$	001	UPPER RESPIRATORY TRACT INFECTION	Dose 1	7	3	Mild	Unlikely related	Recovered/ resolved
		001	GASTROENTERITIS	Dose 1	25	6	Moderate	Unlikely related	Recovered/ resolved
		001	FEVER	Dose 1	28	3	Mild	Unlikely related	Recovered/ resolved
		001	MALARIA	Dose 3	14	4	Mild	Unlikely related	Recovered/ resolved
		005	CUT WOUND	Dose 1	20	15	Moderate	Unlikely related	Recovered/ resolved
		009	MALARIA	Dose 3	14	7	Mild	Unlikely related	Recovered/ resolved
		010	MALARIA	Dose 3	14	4	Mild	Unlikely related	Recovered/ resolved
1a	CHMI Control	163	COMMON COLD	CHMI 1	14	2	Mild	Unlikely related	Recovered/ resolved
1b	$1.8x10^6$	014	VITAMIN C DEFICIENCY	Dose 1	19	7	Mild	Unlikely related	Recovered/ resolved

Group	Dose	Volunteer ID	Adverse Event	Associated with Dose #	# of Days Post Associated Dose	Duration (days)	Severity	Relationship to Study Product	Outcome
	$1.8x10^{6}$	015	OTITIS MEDIA	Dose 2	1	4	Mild	Unlikely related	Recovered/ resolved
2a	$9x10^{5}$	019	VAGINAL CANDIDIASIS	Dose 3	6	18	Mild	Unlikely related	Recovered/ resolved
		049	UPPER RESPIRATORY TRACT INFECTION	Dose 2	6	5	Mild	Unlikely related	Recovered/ resolved
2a	Placebo	017	ABDOMINAL DISCOMFORT	Dose 1	6	3	Mild	Unlikely related	Recovered/ resolved
2b	$1.8x10^{6}$	096	EYE STRAIN	Dose 1	1	1	Moderate	Unlikely related	Recovered/ resolved
		098	UPPER RESPIRATORY TRACT INFECTION	Dose 1	41	6	Mild	Unlikely related	Recovered/ resolved
		107	UPPER RESPIRATORY TRACT INFECTION	Dose 1	20	4	Mild	Unlikely related	Recovered/ resolved
2b	Placebo	074	FEVER	Dose 3	18	2	Mild	Unlikely related	Recovered/ resolved
		101	IRREGULAR MENSTRUATION	Dose 2	23	3	Mild	Unlikely related	Recovered/ resolved

Group	Dose	Volunteer ID	Adverse Event	Associated with Dose #	# of Days Post Associated Dose	Duration (days)	Severity	Relationship to Study Product	Outcome
3b	$1.8x10^6$	081	FEVER	Dose 1	17	2	Mild	Unlikely related	Recovered/ resolved
3b	Placebo	079	CONSTIPATION	Dose 2	14	3	Mild	Unlikely related	Recovered/ resolved
		093	TONSILLITIS	Dose 2	13	3	Mild	Unlikely related	Recovered/ resolved
4a	$4.5x10^5$	052	ACUTE OTITIS MEDIA	Dose 2	17	5	Mild	Unlikely related	Recovered/ resolved
		057	SCALP ABSCESS	Dose 1	4	6	Mild	Unlikely related	Recovered/ resolved
		057	IMPETIGO	Dose 2	14	6	Mild	Unlikely related	Recovered/ resolved
		057	UPPER RESPIRATORY TRACT INFECTION	Dose 2	19	6	Mild	Unlikely related	Recovered/ resolved
		059	GASTRITIS	Dose 1	10	2	Mild	Unlikely related	Recovered/ resolved
		059	ABDOMINAL DISCOMFORT	Dose 1	14	2	Mild	Unlikely related	Recovered/ resolved

Group	Dose	Volunteer ID	Adverse Event	Associated with Dose #	# of Days Post Associated Dose	Duration (days)	Severity	Relationship to Study Product	Outcome
		059	DYSURIA	Dose 1	14	2	Mild	Unlikely related	Recovered/ resolved
		090	UPPER RESPIRATORY TRACT INFECTION	Dose 1	14	4	Mild	Unlikely related	Recovered/ resolved
		090	MULTIPLE INJURIES SECONDARY TO MOTORCYCLE ACCIDENT*	Dose 2	53	9	Not graded	Unrelated	Recovered/ resolved
4a	Placebo	063	GASTROENTERITIS	Dose 2	22	4	Mild	Unlikely related	Recovered/ resolved
		063	PNEUMONIA	Dose 2	50	5	Mild	Unlikely related	Recovered/ resolved
		067	FEVER	Dose 1	14	2	Mild	Possibly related	Recovered/ resolved
		067	FEVER	Dose 1	19	2	Mild	Possibly related	Recovered/ resolved
		067	PNEUMONIA	Dose 2	27	6	Mild	Unlikely related	Recovered/ resolved

Group	Dose	Volunteer ID	Adverse Event	Associated with Dose #	# of Days Post Associated Dose	Duration (days)	Severity	Relationship to Study Product	Outcome
		075	INFECTED WOUND	Dose 2	18	12	Mild	Unlikely related	Recovered/ resolved
4b	$9x10^5$	064	SCALP ABSCESS	Dose 2	8	4	Mild	Unlikely related	Recovered/ resolved
		064	PNEUMONIA	Dose 2	9	4	Mild	Unlikely related	Recovered/ resolved
4b	Placebo	145	FOOD POISONING	Dose 1	19	4	Mild	Unlikely related	Recovered/ resolved
5b	$4.5x10^5$	117	ARM ABSCESS	Dose 2	12	8	Mild	Unlikely related	Recovered/ resolved
		129	PNEUMONIA	Dose 3	8	5	Mild	Unlikely related	Recovered/ resolved
5b	Placebo	125	SEPTICAEMIA	Dose 3	12	5	Mild	Unlikely related	Recovered/ resolved
5c	$9x10^5$	151	PNEUMONIA	Dose 2	23	5	Mild	Unlikely related	Recovered/ resolved
		151	PNEUMONIA	Dose 3	7	7	Mild	Unlikely related	Recovered/ resolved
		151	URINARY TRACT INFECTION	Dose 3	7	7	Mild	Unlikely related	Recovered/ resolved

Group	Dose	Volunteer ID	Adverse Event	Associated with Dose #	# of Days Post Associated Dose	Duration (days)	Severity	Relationship to Study Product	Outcome
		155	URINARY TRACT INFECTION	Dose 2	22	70	Mild	Unlikely related	Recovered/ resolved

Table S7. Natural Infections of malaria parasites identified by retrospective qPCR during the BSPZV2 vaccination period:

Subject	Date	Visit	qPCR [par/uL]	Species	TBS
**001G1a**	12/21/2015	Pre-vaccination 1	negative	-	negative
	02/13/2016	Pre-vaccination 2	35.29	*P. falciparum*	negative
	04/13/2016	Pre-vaccination 3	1.74	*P. falciparum*	negative
	04/26/2016	Post-vaccination follow-up	0.23	*P. falciparum*	negative
	07/01/2016	Pre-CHMI	negative	-	negative
**009G1a**	12/22/2015	Pre-vaccination 1	0.2	*P. malariae*	negative
	02/13/2016	Pre-vaccination 2	0.25	*P. malariae*	negative
	04/13/2016	Pre-vaccination 3	0.06	*P. malariae*	negative
	04/28/2016	Post-vaccination follow-up	0.59	*P. malariae*	negative
	07/01/2016	Pre-CHMI	negative	-	negative
**010G1a**	12/22/2015	Pre-vaccination 1	4.22	*P. malariae*	negative
	02/13/2016	Pre-vaccination 2	1.89	*P. malariae*	negative
	04/13/2016	Pre-vaccination 3	1.2	*P. malariae*	negative
	04/28/2016	Post-vaccination follow-up	0.97	*P. malariae*	negative
	07/10/2016	Pre-CHMI	negative	-	negative
**115G5b**	02/18/2016	Pre-vaccination 1	164.93	*P. falciparum*	negative
	04/12/2016	Pre-vaccination 2	204.45	*P. falciparum*	positive
	07/01/2016	Pre-vaccination 3	negative	-	negative
	07/31/2016	Post-vaccination follow-up	negative	-	negative
	08/28/2016	Post-vaccination follow-up	negative	-	negative

Volunteers 001G1a, 009G1a and 010G1a were treated with artesunate-amodiaquine daily x 3 days beginning 05/02/2016. Volunteer 115G5b was treated with 6 doses artemether-lumefantrine beginning 04/13/2016 (earlier than the other three, because the parasitemia was identified in real time prior to the second immunization due to a positive TBS).

Table S8: *P. falciparum* field isolate genotyping and comparison to NF54 vaccine strain

Method	Marker	NF54	001G1a	115G5b
MSP genotyping	MSP-1	272 bp (K1 family)	191 bp (RO33 family)	232 bp (K1 family)
	MSP-2	447 bp (3D7 family)	364 bp (FC27 family)	487 bp (3D7 family)
Microsatellite genotyping	PFPK2	169 bp	190 bp	163 bp
	TA81	122 bp	131 bp	125 bp
	TA87	100 bp	109 bp	100 bp
	2490	84 bp	93 bp	87 bp
	TA1	186 bp	165 bp	177 bp
	TA109	174 bp	201 bp	180 bp
	PFG377	101 bp	95 bp	98 bp

Table S9a. Laboratory abnormalities by group, grade 1.

	Group 1 (18-45 years)				Group 2 (11-17 years)			Group 3 (6-10 years)		
**Lab parameter**	**9x10^5 (N=6)**	**1.8x10^6 (N=6)**	**Placebo (N=6)**	**CHMI Controls (N=12)**	**9x10^5 (N=6)**	**1.8x10^6 (N=6)**	**Placebo (N=6)**	**9x10^5 (N=6)**	**1.8x10^6 (N=6)**	**Placebo (N=6)**
Leukopenia	0 (0.0)	2 (33.3)	1 (16.7)	3 (25.0)	0 (0.0)	1 (16.7)	0 (0.0)	2 (33.3)	2 (33.3)	1 (16.7)
Neutropenia	0 (0.0)	0 (0.0)	1 (16.7)	0 (0.0)	0 (0.0)	0 (0.0)	1 (16.7)	0 (0.0)	3 (50.0)	0 (0.0)
Lymphopenia	0 (0.0)	3 (50.0)	2 (33.3)	2 (16.7)	2 (33.3)	1 (16.7)	0 (0.0)	2 (33.3)	2 (33.3)	2 (33.3)
Eosinophilia	0 (0.0)	0 (0.0)	0 (0.0)	1 (8.3)	0 (0.0)	0 (0.0)	0 (0.0)	0 (0.0)	0 (0.0)	0 (0.0)
Decreased hemoglobin	1 (16.7)	3 (50.0)	2 (33.3)	2 (16.7)	3 (50.0)	3 (50.0)	3 (50.0)	0 (0.0)	0 (0.0)	2 (33.3)
Thrombocytopenia	1 (16.7)	0 (0.0)	0 (0.0)	1 (8.3)	0 (0.0)	0 (0.0)	0 (0.0)	0 (0.0)	0 (0.0)	0 (0.0)
Elevated creatinine	3 (50.0)	0 (0.0)	2 (33.3)	3 (25.0)	0 (0.0)	0 (0.0)	0 (0.0)	2 (33.3)	2 (33.3)	0 (0.0)
Elevated total bilirubin	0 (0.0)	0 (0.0)	1 (16.7)	0 (0.0)	0 (0.0)	0 (0.0)	0 (0.0)	2 (33.3)	0 (0.0)	0 (0.0)
Elevated ALT	2 (33.3)	1 (16.7)	0 (0.0)	0 (0.0)	0 (0.0)	0 (0.0)	0 (0.0)	0 (0.0)	0 (0.0)	0 (0.0)
Elevated AST	0 (0.0)	1 (16.7)	1 (16.7)	1 (8.3)	0 (0.0)	0 (0.0)	0 (0.0)	0 (0.0)	0 (0.0)	1 (16.7)

	Group 4 (1-5 years)			Group 5 (6-11 months)			
**Lab parameter**	**$4.5x10^5$ (N=6)**	**$9x10^5$ (N=6)**	**Placebo (N=6)**	**$2.7x10^5$ (N=3)**	**$4.5x10^5$ (N=6)**	**$9x10^5$ (N=6)**	**Placebo (N=6)**
Leukopenia	2 (33.3)	0 (0.0)	2 (33.3)	0 (0.0)	0 (0.0)	0 (0.0)	0 (0.0)
Neutropenia	2 (33.3)	0 (0.0)	1 (16.7)	0 (0.0)	1 (16.7)	3 (50.0)	1 (16.7)
Lymphopenia	1 (16.7)	0 (0.0)	0 (0.0)	0 (0.0)	0 (0.0)	0 (0.0)	0 (0.0)
Eosinophilia	1 (16.7)	0 (0.0)	0 (0.0)	0 (0.0)	0 (0.0)	1 (16.7)	0 (0.0)
Decreased hemoglobin	0 (0.0)	0 (0.0)	1 (16.7)	0 (0.0)	3 (50.0)	1 (16.7)	0 (0.0)
Thrombocytopenia	1 (16.7)	0 (0.0)	0 (0.0)	0 (0.0)	0 (0.0)	0 (0.0)	0 (0.0)
Elevated creatinine	0 (0.0)	0 (0.0)	0 (0.0)	0 (0.0)	1 (16.7)	1 (16.7)	1 (16.7)
Elevated total bilirubin	1 (16.7)	0 (0.0)	1 (16.7)	0 (0.0)	0 (0.0)	0 (0.0)	0 (0.0)
Elevated ALT	1 (16.7)	0 (0.0)	0 (0.0)	0 (0.0)	1 (16.7)	0 (0.0)	1 (16.7)
Elevated AST	0 (0.0)	0 (0.0)	0 (0.0)	0 (0.0)	1 (16.7)	0 (0.0)	1 (16.7)

Table shows the number (%) of subjects that had a grade 1 abnormal lab abnormality post-vaccination or CHMI.

Table S9b. Laboratory abnormalities by group, grade 2 and higher.

	Group 1 (18-45 years)				Group 2 (11-17 years)			Group 3 (6-10 years)		
**Lab parameter**	**$9x10^5$ (N=6)**	**$1.8x10^6$ (N=6)**	**Placebo (N=6)**	**CHMI Controls (N=12)**	**$9x10^5$ (N=6)**	**$1.8x10^6$ (N=6)**	**Placebo (N=6)**	**$9x10^5$ (N=6)**	**$1.8x10^6$ (N=6)**	**Placebo (N=6)**
Leukopenia	0 (0.0)	0 (0.0))	0 (0.0)	0 (0.0)	1 (16.7)	0 (0.0)	0 (0.0)	0 (0.0)	0 (0.0)	0 (0.0)
Neutropenia	0 (0.0)	0 (0.0)	0 (0.0)	0 (0.0)	1 (16.7)	0 (0.0)	0 (0.0)	0 (0.0)	0 (0.0)	0 (0.0)
Lymphopenia	2 (33.3)	1 (16.7)*	1 (16.7)	1 (8.3)	0 (0.0)	0 (0.0)	0 (0.0)	0 (0.0)	0 (0.0)	0 (0.0)
Eosinophilia	1 (16.7)	1 (16.7)	0 (0.0)	0 (0.0)	2 (33.3)	2 (33.3)	0 (0.0)	0 (0.0)	0 (0.0)	0 (0.0)
Decreased hemoglobin	0 (0.0)	1 (16.7)	0 (0.0)	0 (0.0)	1 (16.7)	0 (0.0)	1 (16.7)	0 (0.0)	0 (0.0)	1 (16.7)
Thrombocytopenia	0 (0.0)	0 (0.0)	0 (0.0)	0 (0.0)	0 (0.0)	0 (0.0)	0 (0.0)	0 (0.0)	0 (0.0)	0 (0.0)
Elevated creatinine	0 (0.0))	0 (0.0)	0 (0.0)	0 (0.0)	0 (0.0)	0 (0.0)	0 (0.0)	0 (0.0)	0 (0.0)	0 (0.0)
Elevated total bilirubin	0 (0.0)	0 (0.0)	0 (0.0)	0 (0.0)	0 (0.0)	0 (0.0)	0 (0.0)	0 (0.0)	0 (0.0)	0 (0.0)
Elevated ALT	0 (0.0)	1 (16.7)	1 (16.7)	0 (0.0)	0 (0.0)	0 (0.0)	0 (0.0)	0 (0.0)	0 (0.0)	1 (16.7)
Elevated AST	1 (16.7)	0 (0.0)	0 (0.0)	0 (0.0)	0 (0.0)	0 (0.0)	0 (0.0)	0 (0.0)	0 (0.0)	0 (0.0)

	Group 4 (1-5 years)			Group 5 (6-11 months)			
**Lab parameter**	**$4.5x10^5$ (N=6)**	**$9x10^5$ (N=6)**	**Placebo (N=6)**	**$2.7x10^5$ (N=3)**	**$4.5x10^5$ (N=6)**	**$9x10^5$ (N=6)**	**Placebo (N=6)**
Leukopenia	0 (0.0)	0 (0.0)	0 (0.0)	0 (0.0)	0 (0.0)	0 (0.0)	0 (0.0)
Neutropenia	0 (0.0)	0 (0.0)	0 (0.0)	0 (0.0)	2 (33.3)*	0 (0.0)	0 (0.0)
Lymphopenia	0 (0.0)	0 (0.0)	3 (50.0)	0 (0.0)	0 (0.0)	0 (0.0)	0 (0.0)
Eosinophilia	0 (0.0)	0 (0.0)	0 (0.0)	0 (0.0)	0 (0.0)	0 (0.0)	0 (0.0)
Decreased hemoglobin	0 (0.0)	0 (0.0)	0 (0.0)	0 (0.0)	0 (0.0)	1 (16.7)	0 (0.0)
Thrombocytopenia	1 (16.7)	0 (0.0)	0 (0.0)	0 (0.0)	0 (0.0)	0 (0.0)	0 (0.0)
Elevated creatinine	0 (0.0)	0 (0.0)	0 (0.0)	0 (0.0)	0 (0.0)	0 (0.0)	0 (0.0)
Elevated total bilirubin	0 (0.0)	0 (0.0)	0 (0.0)	0 (0.0)	0 (0.0)	0 (0.0)	0 (0.0)
Elevated ALT	0 (0.0)	0 (0.0)	0 (0.0)	0 (0.0)	0 (0.0)	0 (0.0)	0 (0.0)
Elevated AST	0 (0.0)	0 (0.0)	0 (0.0)	0 (0.0)	0 (0.0)	0 (0.0)	0 (0.0)

Table shows the number (%) of subjects that had a grade 2 or higher abnormal lab abnormality post-vaccination or CHMI.

* Adverse events that were Grade 3 (one subject each).

Table S10. Antibody data for all vaccinated volunteers in BSPZV2, as measured by PfCSP ELISA, aIFA, and aISI assays. All out-of-range values and zeroes are reported as 1.

Group (PfSPZ/ Dose)	ID	Age	ELISA PfCSP OD 1.0				aIFA AFU 2x10^5				aISI Reciprocal serum dilution for 80% inhibition			
			Pre-Immune	2 weeks post 3rd dose	Net (Post-Pre)	Ratio (Post/Pre)	Pre-Immune	2 weeks post 3rd dose	Net (Post-Pre)	Ratio (Post/Pre)	Pre-Immune	2 weeks post 3rd dose	Net (Post-Pre)	Ratio (Post/Pre)
1a (9.0x10^5)	001	22 y	274	1958	1684	7.15	32	803	771	25.28	1.00	1.00	0.00	1.00
	005	21 y	238	1030	792	4.33	1	245	244	244.57	1.00	18.05	17.05	18.05
	007	35 y	1	10,045	10,044	10,045.00	1	10,214	10,213	10,213.73	4.26	1.00	-3.26	0.23
	008	21 y	215	1334	1119	6.20	1	348	347	347.99	1.00	14.48	13.48	14.48
	009	20 y	233	689	456	2.96	75	279	204	3.73	4.35	10.20	5.85	2.35
	010	22 y	1006	4498	3492	4.47	113	981	868	8.69	1.00	3.19	2.19	3.19
	**Median**	**22 y**	**236**	**1646**	**1402**	**5.34**	**16**	**576**	**559**	**134.92**	**1.00**	**6.70**	**4.02**	**2.77**
1b (1.8x10^6)	004	20 y	125	8128	8003	65.02	1	7474	7473	7474.07	1.00	25.78	24.78	25.78
	012	27 y	175	27,828	27,653	159.02	41	6447	6406	157.80	3.99	23.52	19.53	5.90
	014	23 y	285	1716	1431	6.02	122	385	263	3.17	1.00	6.35	5.35	6.35
	015	24 y	1	29,872	29,871	29,872.00	8	12,397	12,389	1609.71	1.00	21.75	20.75	21.75
	022	33 y	181	8603	8422	47.53	44	4207	4163	95.77	2.95	22.00	19.05	7.45
	034	18 y	92	5701	5609	61.97	13	2106	2093	160.18	7.24	41.21	33.97	5.69
	**Median**	**24 y**	**150**	**8366**	**8213**	**63.50**	**27**	**5327**	**5284**	**158.99**	**1.98**	**22.76**	**20.14**	**6.90**
2a (9.0x10^5)	019	11 y	26	3538	3512	136.08	192	5009	4817	26.09	1.00	29.56	28.56	29.56
	027	11 y	22	16,243	16,221	738.32	1	15,232	15,231	15,232.17	3.16	1.54	-1.62	0.49
	028	13 y	282	4104	3822	14.55	111	1253	1142	11.27	4.88	9.72	4.84	1.99
	030	13 y	1	2682	2681	2682.00	1	6292	6291	6292.41	1.00	15.47	14.47	15.47
	031	13 y	55	6312	6257	114.76	74	2895	2821	39.26	1.00	21.14	20.14	21.14
	049	11 y	18	12,186	12,168	677.00	1	7464	7463	7463.96	1.69	14.24	12.54	8.41
	**Median**	**12 y**	**18**	**5208**	**5040**	**406.54**	**37**	**5651**	**5554**	**3165.83**	**1.35**	**14.86**	**13.51**	**11.94**
2b (1.8x10^6)	096	11 y	157	26,421	26,264	168.29	1	26,839	26,838	26,838.52	3.66	30.64	26.98	8.38
	097	13 y	1	75,038	75,037	75,038.00	1	3908	3907	3908.16	1.00	79.21	78.21	79.21
	098	15 y	135	26,075	25,940	193.15	1	6046	6045	6046.14	4.29	49.56	45.28	11.57
	102	13 y	1	17,700	17,699	17,700.00	1	18,514	18,513	18,513.60	1.00	37.83	36.83	37.83
	104	14 y	33	31,250	31,217	946.97	37	4129	4092	112.53	2.93	46.15	43.22	15.73
	107	13 y	23	12,143	12,120	527.96	13	19,753	19,739	1490.34	1.00	40.38	39.38	40.38
	**Median**	**13 y**	**28**	**26,248**	**26,102**	**737.46**	**1**	**12,280**	**12,279**	**4977.15**	**1.97**	**43.27**	**41.30**	**26.78**
3a (9.0x10^5)	035	10 y	51	3160	3109	61.96	1	1968	1967	1967.85	2.73	11.50	8.77	4.21
	036	7 y	42	1570	1528	37.38	1	1377	1376	1376.96	1.00	15.96	14.96	15.96
	039	9 y	25	2664	2639	106.56	1	2757	2756	2756.69	1.00	1.00	0.00	1.00
	040	7 y	7	2057	2050	293.86	13	1199	1187	93.85	1.00	7.54	6.54	7.54
	048	8 y	16	9537	9521	596.06	1	9786	9785	9786.41	1.00	42.64	41.64	42.64
	050	6 y	60	2534	2474	42.23	1	3370	3369	3370.24	1.00	8.10	7.10	8.10
	**Median**	**8 y**	**34**	**2599**	**2557**	**84.26**	**1**	**2362**	**2361**	**2362.27**	**1.00**	**9.80**	**7.94**	**7.82**
3b (1.8x10^6)	080	6 y	18	76,513	76,495	4250.72	1	13,709	13,708	13,708.78	1.84	48.38	46.54	26.32
	081	8 y	1	928	31,544	31,545.00	1	20,832	20,831	20,831.51	1.00	63.34	62.34	63.34

	083	9 y	68	1694	1626	24.91	1	51,052	51,051	51,052.32	1.00	1.00	0.00	1.00
	084	7 y	1	26,778	26,777	26,778.00	9	76,829	76,821	8778.57	2.75	27.29	24.54	9.92
	092	7 y	1	38,042	38,041	38,042.00	1	5840	5839	5840.03	1.70	18.85	17.15	11.08
	100	10 y	25	43,479	43,455	1811.63	1	19,369	19,368	19,369.25	2.27	24.48	22.21	10.79
	**Median**	**8 y**	**10**	**34,794**	**34,793**	**15,515.36**	**1**	**20,100**	**20,099**	**16,539.02**	**1.77**	**25.89**	**23.38**	**10.94**
**4a (4.5x10^5)**	052	3 y	55	20,912	20,857	380.22	43	100	58	2.34	1.37	8.06	6.69	5.88
	057	3 y	66	1493	1497	22.62	1	5244	5243	5244.21	1.97	7.86	5.89	4.00
	059	4 y	23	9163	9140	398.39	82	23,040	22,958	282.24	3.19	10.45	7.26	3.28
	060	5 y	68	1353	1285	19.90	1	1094	1093	1093.77	1.00	1.00	0.00	1.00
	070	3 y	21	10,729	10,708	510.90	73	34,965	34,892	480.16	2.59	21.12	18.53	8.16
	090	2 y	1	7721	7720	7721.00	48	3750	3702	77.61	1.00	12.79	11.79	12.79
	**Median**	**3 y**	**39**	**8442**	**8430**	**389.30**	**46**	**4497**	**4472**	**381.20**	**1.67**	**9.26**	**6.98**	**4.94**
**4b (9.0x10^5)**	051	4 y	1	10,516	10,515	10,516.00	55	10,231	10,177	187.28	4.56	9.63	5.07	2.11
	058	5 y	62	6032	5970	97.29	30	101	71	3.41	1.18	16.96	15.77	14.31
	064	1 y	25	12,847	12,822	513.88	34	1	-33	0.03	2.84	24.36	21.52	8.58
	065	1 y	40	13,206	13,166	330.15	1	7461	7460	7461.09	1.00	24.95	23.95	24.95
	144	4 y	1	93	92	93.00	71	3752	3681	52.51	1.00	3.34	2.34	3.34
	146	1 y	192	6338	6146	33.01	9	11,810	11,801	1331.91	3.03	16.18	13.14	5.34
	**Median**	**3 y**	**33**	**8427**	**8331**	**213.72**	**32**	**5607**	**5570**	**119.90**	**2.01**	**16.57**	**14.46**	**6.96**
**5b (4.5x10^5)**	089	7 mo	17	158	141	9.29	39	101	62	2.60	1.00	2.77	1.77	2.77
	114	7 mo	1	834	833	834.00	1	313	312	313.42	1.00	4.19	3.19	4.19
	117	7 mo	199	1257	1058	6.32	1	270	269	270.12	1.00	6.23	5.23	6.23
	118	8 mo	315	6158	5843	19.55	1	1991	1990	1991.17	1.74	19.40	17.66	11.15
	129	8 mo	188	714	526	3.80	1	111	110	111.04	1.00	6.62	5.62	6.62
	134	8 mo	10	4207	4197	420.70	1	2572	2571	2572.34	1.00	12.60	11.60	12.60
	**Median**	**8 mo**	**103**	**1046**	**946**	**14.42**	**1**	**292**	**291**	**291.77**	**1.00**	**6.43**	**5.43**	**6.43**
**5c (9.0x10^5)**	126	9 mo	19	20,493	20,474	1078.58	1	11,315	11,314	11,315.09	2.08	66.81	64.73	32.16
	131	9 mo	15	14,969	14,954	997.93	5	1	-4	0.20	1.00	14.66	13.66	14.66
	133	8 mo	1	74	73	74.00	68	20,148	20,079	294.28	1.94	4.45	2.51	2.29
	151	9 mo	49	37,788	37,739	771.18	1	6242	6241	6241.57	1.00	96.68	95.68	96.68
	155	11 mo	1159	14,874	13,715	12.83	32	6053	6021	189.99	1.00	15.18	14.18	15.18
	157	11 mo	1	2267	2266	2267.00	1	606	605	605.80	1.00	19.49	18.49	19.49
	**Median**	**9 mo**	**17**	**14,922**	**14,335**	**884.56**	**3**	**6147**	**6131**	**450.04**	**1.00**	**17.34**	**16.34**	**17.34**

Table S11. Immunological data for all control volunteers in BSPZV2, as measured in PfCSP ELISA, aIFA, and aISI assays. All out-of-range values and zeroes are reported as 1.

Group (PfSPZ/ Dose)	ID	Age	ELISA PfCSP OD 1.0				aIFA AFU 2x10^5				aISI Reciprocal serum dilution for 80% inhibition			
			Pre-Immune	2 weeks post 3rd dose	Net (Post-Pre)	Ratio (Post/Pre)	Pre-Immune	2 weeks post 3rd dose	Net (Post-Pre)	Ratio (Post/Pre)	Pre-Immune	2 weeks post 3rd dose	Net (Post-Pre)	Ratio (Post/Pre)
**1a (9.0x10^5)**	002	36 y	102	102	0	1.00	1.00	1.00	0.00	1.00	1.00	1.00	0.00	1.00
	003	31 y	68	42	-26	0.62	1.00	1.00	0.00	1.00	9.84	8.54	-1.30	0.87
	006	19 y	46	38	-8	0.83	1.00	1.00	0.00	1.00	1.00	1.00	0.00	1.00
	**Median**	**31 y**	**68**	**42**	**-8**	**0.83**	**1.00**	**1.00**	**0.00**	**1.00**	**1.00**	**1.00**	**0.00**	**1.00**
**1b (1.8x10^6)**	011	28 y	245	311	66	1.27	1.00	1.00	0.00	1.00	1.00	1.00	0.00	1.00
	013	38 y	284	302	18	1.06	50.37	48.90	-1.47	0.97	1.00	11.17	**10.17**	**11.17**
	023	20 y	24	16	-8	0.67	1.00	1.00	0.00	1.00	1.00	1.00	0.00	1.00
	**Median**	**28 y**	**245**	**302**	**18**	**1.06**	**1.00**	**1.00**	**0.00**	**1.00**	**1.00**	**1.00**	**0.00**	**1.00**
**2a (9.0x10^5)**	016	14 y	9	1	-8	0.11	1.00	1.00	0.00	1.00	1.00	1.00	0.00	1.00
	017	11 y	18	16	-2	0.89	1.00	1.00	0.00	1.00	5.25	5.08	0.17	1.03
	029	13 y	200	407	207	2.04	1.00	1.00	0.00	1.00	1.00	4.21	3.21	4.21
	**Median**	**13 y**	**18**	**16**	**-2**	**0.89**	**1.00**	**1.00**	**0.00**	**1.00**	**1.00**	**4.21**	**0.17**	**1.03**
**2b (1.8x10^6)**	074	11 y	736	444	-292	0.60	3.10	1.00	-2.10	0.32	1.00	1.00	0.00	1.00
	094	11 y	31	54	23	1.74	1.00	1.00	0.00	1.00	3.46	4.91	1.45	1.42
	101	13 y	43	63	20	1.47	1.00	1.00	0.00	1.00	3.31	15.03	**11.72**	**4.54**
	**Median**	**11 y**	**43**	**63**	**20**	**1.47**	**1.00**	**1.00**	**0.00**	**1.00**	**3.31**	**4.91**	**1.45**	**1.42**
**3a (9.0x10^5)**	042	7 y	22	57	35	2.59	1.00	1.00	0.00	1.00	3.28	10.06	6.77	3.06
	045	8 y	1	39	38	39.00	1.00	1.00	0.00	1.00	1.00	1.00	0.00	1.00
	047	9 y	10	1	-9	0.10	1.00	1.00	0.00	1.00	4.07	5.81	1.74	1.43
	**Median**	**8 y**	**10**	**39**	**35**	**2.59**	**1.00**	**1.00**	**0.00**	**1.00**	**3.28**	**5.81**	**1.74**	**1.43**
**3b (1.8x10^6)**	076	6 y	21	21	0	1.00	1.00	1.00	0.00	1.00	7.36	11.85	4.48	1.61
	079	10 y	27	25	-2	0.93	77.04	33.49	-43.55	0.43	1.00	2.92	1.92	2.92
	093	7 y	203	241	38	1.19	1.00	1.00	0.00	1.00	1.00	3.62	2.62	3.62
	**Median**	**7 y**	**27**	**25**	**0**	**1.00**	**1.00**	**1.00**	**0.00**	**1.00**	**1.00**	**3.62**	**2.62**	**2.92**
**4a (4.5x10^5)**	063	2 y	21	27	6	1.29	1.00	1.00	0.00	1.00	2.81	3.59	0.79	1.28
	067	3 y	12	29	17	2.42	1.00	1.00	0.00	1.00	1.00	7.95	6.95	7.95
	075	4 y	54	49	-5	0.91	46.63	1.00	-45.63	0.02	5.89	1.00	-5.89	0.00
	**Median**	**3 y**	**21**	**29**	**6**	**1.29**	**1.00**	**1.00**	**0.00**	**1.00**	**2.81**	**3.59**	**0.79**	**1.28**
**4b (9.0x10^5)**	085	2 y	167	22	-145	0.13	1.00	1.00	0.00	1.00	27.90	10.21	-17.69	0.37
	143	2 y	1	46	45	46.00	1.00	1.00	0.00	1.00	1.00	1.00	0.00	1.00
	145	5 y	34	26	-8	0.76	1.00	1.00	0.00	1.00	1.00	5.11	4.11	5.11
	**Median**	**2 y**	**34**	**26**	**-8**	**0.76**	**1.00**	**1.00**	**0.00**	**1.00**	**1.00**	**5.11**	**0.00**	**1.00**
**5b (4.5x10^5)**	115	6 mo	54	34	-20	0.63	1.00	1.00	0.00	1.00	1.00	2.91	1.91	2.91
	124	11 mo	13	22	9	1.69	1.00	1.00	0.00	1.00	1.00	1.00	0.00	1.00
	125	9 mo	1	60	**59**	**60.00**	1.00	1.00	0.00	1.00	1.00	1.00	0.00	1.00
	**Median**	**9 mo**	**13**	**34**	**9**	**1.69**	**1.00**	**1.00**	**0.00**	**1.00**	**1.00**	**1.00**	**0.00**	**1.00**

**5c** **(9.0x10^5)**	136	10 mo	21	1	-20	0.05	1.00	1.00	0.00	1.00	1.00	1.00	0.00	1.00
	147	11 mo	19	28	9	1.47	1.00	1.00	0.00	1.00	1.00	1.00	0.00	1.00
	154	11 mo	26	91	**65**	**3.50**	1.00	1.00	0.00	1.00	1.00	1.00	0.00	1.00
	**Median**	**11 mo**	**21**	**28**	**9**	**1.47**	**1.00**	**1.00**	**0.00**	**1.00**	**1.00**	**1.00**	**0.00**	**1.00**

Table S12. Percent of memory CD4 T cells in the blood expressing IFNγ, IL-2, or TNFα at pre-immunization or 2 weeks after the first and third doses of PfSPZ Vaccine at a dose of $9.0x10^5$ PfSPZ three times at 8 week intervals. Results are the percentage of cytokine-producing cells after incubation with PfSPZ minus the percentage of cytokine-producing cells after incubation with vaccine diluent (medium with 1% human serum albumin).

Group (PfSPZ/ Dose)	ID	Age	Individual CD4 T Cell Responses (% $IFN\gamma^+$, $IL\text{-}2^+$, or $TNF\alpha^+$)		
			Pre-Immune	2 weeks post 1st dose	2 weeks post 3rd dose
1a (18-45 years) ($9.0x10^5$)	001	22 y	0.031	0.220	0.158
	005	21 y	-0.021	-0.034	0.096
	007	35 y	0.191	1.346	0.260
	008	21 y	0.045	0.094	0.116
	009	20 y	-0.030	0.002	0.054
	010	22 y	0.258	1.194	0.449
	**Median**	**22 y**	**0.038**	**0.157**	**0.137**
2a (11-17 years) ($9.0x10^5$)	019	11 y	0.057	0.605	0.385
	027	11 y	0.025	0.509	0.114
	028	13 y	0.045	0.032	-0.068
	030	13 y	0.013	0.390	0.217
	031	13 y	0.002	-0.005	0.011
	049	11 y	-0.003	0.609	0.534
	**Median**	**12 y**	**0.019**	**0.450**	**0.166**
3a (6-10 years) ($9.0x10^5$)	035	10 y	0.052	0.435	0.211
	036	7 y	0.018	0.534	0.198
	039	9 y	0.039	1.073	0.206
	040	7 y	0.000	0.583	0.121
	048	8 y	0.040	0.396	0.142
	050	6 y	0.282	0.515	0.126
	**Median**	**8 y**	**0.040**	**0.525**	**0.170**
4b (1-5 years) ($9.0x10^5$)	051	4 y	0.074	0.331	0.156
	058	5 y	0.116	0.215	0.288
	064	1 y	-0.118	0.179	0.731
	065	1 y	0.035	0.139	0.275
	144	4 y	0.020	0.048	0.089
	146	1 y	-0.027	-0.075	0.198
	**Median**	**3 y**	**0.028**	**0.159**	**0.237**
5c (6-12 months) ($9.0x10^5$)	126	9 mo	0.016	0.112	0.169
	131	9 mo	0.085	0.370	-0.043
	133	8 mo	-0.073	0.051	0.101
	151	9 mo	0.013	0.068	-0.001
	155	11 mo	0.072	-0.046	0.473
	157	11 mo	-0.138	0.216	0.104
	**Median**	**9 mo**	**0.015**	**0.090**	**0.103**

Table S13. Percent of memory CD4 T cells in the blood expressing IFNγ, IL-2, or TNFα at pre-immunization or 2 weeks after the first and third doses of normal saline (controls). Results are the percentage of cytokine-producing cells after incubation with PfSPZ minus the percentage of cytokine-producing cells after incubation with vaccine diluent (medium with 1% human serum albumin).

Group (PfSPZ/ Dose)	ID	Age	%IFNγ$^+$, IL-2$^+$, or TNFα$^+$ of memory CD4 T cells		
			Pre-Immune	2 weeks post 1st dose	2 weeks post 3rd dose
1a (9.0x10^5)	002	36 y	0.002	0.084	0.068
	003	31 y	0.009	0.028	-0.027
	006	19 y	0.062	-0.31	0.031
	**Median**	31 y	0.009	0.028	0.031
2b (9.0x10^5)	016	14 y	0.187	0.079	0.033
	017	11 y	0.057	0.046	0.006
	029	13 y	0.194	0.104	-0.05
	**Median**	13 y	0.187	0.079	0.006
3a (9.0x10^5)	042	7 y	0.033	0.022	0.037
	045	8 y	0.063	0.061	0.025
	047	9 y	0.035	0.024	0.039
	**Median**	8 y	0.035	0.024	0.037
4b (9.0x10^5)	085	2 y	0.074	0.358	-0.026
	143	2 y	0.331	0.101	-0.014
	145	5 y	0.156	0.214	-0.016
	**Median**	2 y	0.156	0.214	-0.016
5c (9.0x10^5)	136	10 mo	0.022	0.047	0.128
	147	11 mo	0.013	0.024	-0.232
	154	11 mo	0.014	-0.003	0.026
	**Median**	11 mo	0.014	0.024	0.026
